# Integrated genomic analyses in PDX model reveal a cyclin-dependent kinase inhibitor Palbociclib as a novel candidate drug for nasopharyngeal carcinoma

**DOI:** 10.1186/s13046-018-0873-5

**Published:** 2018-09-20

**Authors:** Cheng-Lung Hsu, Kar-Wai Lui, Lang-Ming Chi, Yung-Chia Kuo, Yin-Kai Chao, Chun-Nan Yeh, Li-Yu Lee, Yenlin Huang, Tung-Liang Lin, Mei-Yuan Huang, Yi-Ru Lai, Yuan-Ming Yeh, Hsien-Chi Fan, An-Chi Lin, Yen-Jung Lu, Chia-Hsun Hsieh, Kai-Ping Chang, Ngan-Ming Tsang, Hung-Ming Wang, Alex Y. Chang, Yu-Sun Chang, Hsin-Pai Li

**Affiliations:** 1 Division of Hematology-Oncology, Department of Internal Medicine, Chang Gung Memorial Hospital, Chang Gung University, No.5, Fuxing St., Guishan Dist, Taoyuan City, 333 Lin-Kou Taiwan, Republic of China; 2 Department of Medical Imaging and Intervention, Chang Gung Memorial Hospital, Chang Gung University, No.5, Fuxing St., Guishan Dist, Taoyuan City, 333 Lin-Kou Taiwan, Republic of China; 3 Clinical Proteomics Core Laboratory, Chang Gung Memorial Hospital, No.5, Fuxing St., Guishan Dist, Taoyuan City, 333 Lin-Kou Taiwan, Republic of China; 4 Division of Thoracic and Cardiovascular Surgery, Department of Surgery, Chang Gung Memorial Hospital, No.5, Fuxing St., Guishan Dist, Taoyuan City, 333 Lin-Kou Taiwan, Republic of China; 5 Department of General Surgery, Liver Research Center, Chang Gung Memorial Hospital, Chang Gung University, No.5, Fuxing St., Guishan Dist, Taoyuan City, 333 Lin-Kou Taiwan, Republic of China; 6 Department of Pathology, Chang Gung Memorial Hospital, Chang Gung University, No.5, Fuxing St., Guishan Dist, Taoyuan City, 333 Lin-Kou Taiwan, Republic of China; 7 Department of Microbiology and Immunology, Molecular Medicine Research Center, Chang Gung University, No.259, Wenhua 1st Rd., Guishan Dist., Lin-Kou, Taoyuan, 333 Taiwan, Republic of China; 8 Molecular Medicine Research Center, Chang Gung University, No.259, Wenhua 1st Rd., Guishan Dist, Taoyuan City, 333 Taiwan, Republic of China; 9 ACT Genomics, Co. Ltd., 1F., No.280, Xinhu 2nd Rd., Neihu Dist, Taipei City, 114 Taiwan, Republic of China; 10 Department of Otolaryngology-Head and Neck Surgery, Chang Gung Memorial Hospital, Chang Gung University, No.5, Fuxing St., Guishan Dist, Taoyuan City, 333 Lin-Kou Taiwan, Republic of China; 11 Department of Radiation, Chang Gung Memorial Hospital, Chang Gung University, No.5, Fuxing St., Guishan Dist, Taoyuan City, 333 Lin-Kou Taiwan, Republic of China; 12 Johns Hopkins Singapore International Medical Centre, 11 Jalan Tan Tock Seng, Singapore City, 308433 Singapore

**Keywords:** Nasopharyngeal carcinoma, Patient derived xenograft, EBV, Whole-exome sequencing, CDK4/6 inhibitor, RNA sequencing

## Abstract

**Background:**

Patient-derived xenograft (PDX) tumor model has become a new approach in identifying druggable tumor mutations, screening and evaluating personalized cancer drugs based on the mutated targets.

**Methods:**

We established five nasopharyngeal carcinoma (NPC) PDXs in mouse model. Subsequently, whole-exome sequencing (WES) and genomic mutation analyses were performed to search for genetic alterations for new drug targets. Potential drugs were applied in two NPC PDX mice model to assess their anti-cancer activities. RNA sequencing and transcriptomic analysis were performed in one NPC PDX mice to correlate with the efficacy of the anti-cancer drugs.

**Results:**

A relative high incident rate of copy number variations (CNVs) of cell cycle-associated genes. Among the five NPC-PDXs, three had *cyclin D1* (*CCND1*) amplification while four had cyclin-dependent kinase inhibitor *CDKN2A* deletion. Furthermore, CCND1 overexpression was observed in > 90% FFPE clinical metastatic NPC tumors (87/91) and was associated with poor outcomes. CNV analysis disclosed that plasma *CCND1*/*CDKN2A* ratio is correlated with EBV DNA load in NPC patients’ plasma and could serve as a screening test to select potential CDK4/6 inhibitor treatment candidates. Based on our NPC PDX model and RNA sequencing, Palbociclib, a cyclin-dependent kinase inhibitor, proved to have anti-tumor effects by inducing G1 arrest. One NPC patient with liver metastatic was treated with Palbociclib, had stable disease response and a drop in Epstein Barr virus (EBV) EBV titer.

**Conclusions:**

Our integrated information of sequencing-based genomic studies and tumor transcriptomes with drug treatment in NPC-PDX models provided guidelines for personalized precision treatments and revealed a cyclin-dependent kinase inhibitor Palbociclib as a novel candidate drug for NPC.

**Electronic supplementary material:**

The online version of this article (10.1186/s13046-018-0873-5) contains supplementary material, which is available to authorized users.

## Background

Nasopharyngeal carcinoma (NPC) is prevalent in southeastern Asia [1]. Individual genetic susceptibility, Epstein-Barr virus (EBV) infection, and dietary/chemical carcinogens are involved in the pathogenesis of NPC [2–5]. NPC tumors are characterized as tumors with relatively low single nucleotide mutation but with frequent hypermethylation, amplification of CCND1, deletion of tumor suppressor genes such as CDKN2A and 2B, and other chromosome abnormalities [6]. Genomic studies on NPC mostly involving whole-exome sequencing (WES) and copy number variation (CNV) analyses have aided in elucidating multiple deregulation pathways associated with chromatin modification, cell cycle G1/S transition, ERBB-PI3K signaling and NF-kB signaling [7–10].

To identify precise druggable targets for cancer treatment, patient-derived xenograft (PDX) model serves as a platform connecting basic and clinical research for new treatment validations and biomarker screening [11–13]. Several NPC-PDX studies including ours have demonstrated NPC-PDX model is a feasible mice model to reflect patient’s response to new treatments [14–16]. Integration of genomic studies for actionable target screening and validation of candidate drugs in PDX models could provide effective guidance for personalized/precision medicine [17]. Here, we present a precision oncology approach that combines WES and CNV analyses, NPC-PDX model to test druggable cell cycle-regulated gene and evaluate the efficacy of CDK inhibitor, palbociclib. Furthermore, using cancer patients’ cell-free DNA, we develop PCR-based EBV copy and CNV tests (CCND1 and CDKN2A) to provide drug guidance information.

## Methods

### Drugs

Gemcitabine (GEM), GSK-126, and decitabine (DEC) were purchased from Sigma Chemical Co (St. Louis, MO). Palbociclib (PAL) was acquired from MedChem Express (Monmouth Junction, NJ).

### Cell growth assay and animal studies

NP69 (T antigen immortalized nasopharyngeal epithelial (NP) cells) [18], C666–1 (NPC cell harboring EBV) [19] and HK-1 (NPC cell without EBV) [20] cells were maintained in RPMI containing 10% fetal bovine serum (FBS). Cell growth assay and animal studies were conducted as described in a previous report [16]. All experiments involving laboratory animals followed the Guidelines for Animal Experiments of CGMH and were approved by the Animal Research CGMH.

### Patient participants

Seventeen biopsy-proven NPC patients with local recurrence or distant metastasis were enrolled between July 2013 and June 2016; one hundred thirty nine NPC patient biopsies/FFPE collected between 2002~ 2016 in CGMH; and NPC patient had Palbociclib written informed consent, approved by the Institutional Review Board of CGMH (IRB No.:98-3119C, 101-5065A, 102-5646A3).

### Patient-derived xenograft (PDX)

PDX models were generated according to a previously reported procedure [16]. Briefly, NPC tumor samples were obtained from patients undergoing biopsy or surgical resection. Each sample was immediately cut into small sections, immersed in antibiotic-containing PBS and implanted subcutaneously in the flank regions of anesthetized NOD/SCID mice. After reaching a diameter of ~ 1 cm, the xenograft was excised and sub-implanted into subsequent passage mice. It took 2~ 4 months to passage PDX tumor in mice.

### Drug sensitivity tests in the PDX model

Tests for drug sensitivity were performed as described previously [16]. Please refer to Additional file 1: supplementary materials and methods for detailed protocol.

### Genomic DNA extraction

Genomic DNA was extracted from PDX, FFPE, and plasma cell-free DNA using the QIAamp DNA mini kit, QlAamp DNA FFPE Tissue Kit (Qiagen), and QIAamp DNA blood mini kit (Qiagen), respectively, according to the manufacturer’s instructions. Extracted DNA samples were quantified using a NanoDrop or Qubit™ dsDNA HS Assay Kit (Invitrogen). Genomic DNA integrity was determined with the Fragment Analyzer™ system (Advanced Analytical Technologies, Inc).

### Whole exome sequencing

Whole exome sequencing (WES) was performed on genomic DNA from NPC PDX tumors and their matched peripheral blood from the corresponding NPC patients (a) (NPC PDX-ST, -LN, −LG, -LV) (Macrogen, Korea, using SureSelectXT Lib. Prep. Kit, HiSeq 4000, Illumina) as well as (b) NPC PDX-Bone and PDX-LN (ACTgenomics, Taiwan).

### Identification of somatic mutations from WES data

Fastq files of WES obtained from Macrogen were filtered and (adaptor) trimmed. Sequencing reads from NPC PDX tumors (ST, LN, LG, LV) were aligned and filtered to the mouse reference genome, MM10, using the Burrows-Wheeler Aligner (BWA) tool. The remaining reads of PDX tumors and sequencing reads from matched patients’ peripheral blood were aligned to the human reference genome, hg19, separately using BWA. Variants from both PDX tumor and normal samples were identified using the Genome Analysis Toolkit (GATK) pipeline. GATK Unified Genotyper was used to call SNVs and Indels (Genomics, Taiwan).

### ACTOnco comprehensive Cancer panel sequencing (ACTgenomics)

Genomic DNA (80 ng) was amplified using four pools of 15,992 primer pairs (Ion AmpliSeq Comprehensive Cancer Panel, Life Technologies) targeting all coding exons of the 409 cancer-related genes. Amplicons were ligated with barcoded adaptors using the Ion Amplicon Library Kit (Life Technologies). Barcoded libraries were subsequently conjugated with sequencing beads via emulsion PCR and enriched using the Ion PI™ Hi-Q™ Chef Kit (Life Technologies). The quality and quantity of the amplified library were determined using fragment analyzer (AATI) and Qubit (Invitrogen). Sequencing was performed on the Ion Proton sequencer using the Ion PI™ Chip Kit v3 (Life Technologies) according to the manufacturer’s protocol.

### Analysis of whole exome sequence data

The library was constructed according to Ion AmpliSeq™ Exome RDY library preparation kit (ACTgenomics). Briefly, 50 ng genomic DNA was amplified using 12 pools of primer pairs (Ion AmpliSeq Exome RDY Kit, Life Technologies) to target all coding exons of 18,835 genes (~ 57.7 Mb). Amplicons were ligated with barcoded adapters using the Ion Xpress™ barcode adapters kit (Life Technologies). Barcoded libraries were subsequently conjugated to sequencing beads via emulsion PCR and enriched with the Ion PI™ Hi-Q™ Chef Kit (Life Technologies). The quality and quantity of the amplified library were determined using the fragment analyzer (AATI) and Qubit (Invitrogen). Sequencing was performed on the Ion Proton sequencer using the Ion PI chip (Life Technologies) according to the manufacturer’s protocol (ACTgenomics). Raw reads generated by the sequencer were mapped to the hg19 reference genome using the Ion Torrent Suite (v. 5.0) and coverage depth calculated using Torrent Coverage Analysis plug-in. Single nucleotide variants (SNVs) and short insertion/deletions (INDELs) were identified using the Torrent Variant Caller plug-in (version 5.0). Variant Effect Predictor (VEP) (version 77) was applied to annotate every variant with a database from COSMIC: v.70; dbSNP 138 and 1000 Genomes: phase1. Variant coverage lower than 25 or frequency lower than 5% were filtered. Variants reported in the 1000 Genomes Project Phase 1 with > 1% minor allele frequency (MAF) and those in the ACT Genomics in-house PBMC database were considered polymorphisms.

### Copy number alteration analysis

Amplicons of PDX-B and WBC-B with read counts in the lowest 5th percentile of all detectable amplicons and those with coefficients of variation ≥0.3 were removed. The remaining amplicons from four different pools were normalized to correct the pool design bias. ONCOCNV [21] was applied for normalization of total amplicon number, GC content, length, and technology-related bias, followed by segmentation of the sample with a gene-aware model. The method was additionally used for establishing the baseline of copy number variations from samples in the ACTgenomics in-house PBMC database.

Whole exome sequencing reads [Fastq format, NPC PDX and PBMC (-ST, -LN, −LG, -LV)] were mapped to the human reference genome, hg19, using the FANSe2 algorithm [22] with 5% error tolerance. Uniquely mapped reads were employed for further analysis to avoid ambiguity. Read density was calculated for each gene and each sample as read count divided by exon length. The average read densities of normal karyotype WBC samples were used as the normalization standard. Read density of each gene and each sample was normalized against standard values (Changgong Biotech., Taiwan). Since all the samples were from male patients, normal copy numbers of autosomal chromosomes were set as 2 while those of sex chromosomes X and Y were set as 1. The copy number of each gene and sample was plotted accordingly.

### RNAseq

RNA of PDX samples was extracted using TRIzol (Invitrogen) reagent following the manufacturer’s protocol. The remaining DNA was eliminated by treatment with DNase I as recommended by the manufacturer. Intact PolyA+ mRNA was selected using the NEB Poly(A) mRNA Magnetic Isolation Module (New England Biolabs). mRNA libraries were constructed with the aid of the NEBNext Ultra RNA library prep kit for Illumina (New England Biolabs) following the manufacturer’s protocol. Sequencing was performed on an Illumina HiSeq X Ten sequencer for 150 cycles. The high-quality reads that passed the Illumina filter were subjected to bioinformatics analyses (Changgong Biotech). Sequences were mapped to a combined reference sequence database containing human RefSeq-RNA, mouse RefSeq-RNA and EB virus sequences (NCBI accession: AY961628, DQ279927 and V01555) using the hyper-accurate mapping algorithm FANSe2 [22] in the NGS analysis platform “Chi-Cloud” (http://www.chi-biotech.com). Reads mapped to the mouse reference sequences were discarded and splice variants merged. Gene expression levels were quantified using the RPKM method [23]. Genes with at least 10 reads were considered quantifiable [24]. Differentially expressed genes (DEG) were analyzed using the edgeR package (version 3.12.0) [25] considering at least a 2-fold change and *p* < 0.05. Gene ontology and pathway analyses of DEGs were performed using topGO (version 2.22.0) [26] and KOBAS (version 2.0) [27], respectively.

### Antibodies

One hundred microgram protein lysate per lane was used for Western blot analysis. Antibodies used in this study: RB1 (CusaBio PA003948), RB-P (Cell Signaling 9307), E2F1 (Santa Cruz SC-193), CDK2 (CusaBio PA001533), CDK4 (Santa Cruz SC-23896), CDK6 (Santa Cruz SC-53638), CCND1 (Santa Cruz SC-8396), CCNE2 (Proteintech 11,935–1-AP), CDKN2A (Prosci 4211), CDKN1A (Santa Cruz SC-6246), PCNA (Proteintech 10,205–2-AP) and GAPDH (Santa Cruz FL-335).

### Statistical analysis

Cell line and tumor weight data are presented as means ± SD. Final tumor volumes were compared using two-tailed analysis of variance (ANOVA). Correlations of CNV of RAD52, CCND1 and CCND2A with EBV DNA load were depicted via linear regression. Overall survival was calculated from the time of obtaining tissue for PDX to death, plotted via Kaplan-Meier curves, and compared using the log-rank test. In all analyses, *p*-values were two-tailed and data were considered statistically significant at *p*-values less than 0.05.

## Results

### Establishment of six NPC-PDX lines and analysis of their genomic mutations

Five NPC-PDX lines were successfully established from seventeen biopsy-proven NPC cases with local recurrence/metastasis between July 2013 and June 2016 (Additional file 2: Table S1). The metastatic sites of the NPC-PDX parental tumors included soft tissue (ST), lymph node (LN), lung (LG), liver (LV) and bone. Tumor take rate for PDX-engraftment was ~ 30%. PDX engraftment-positive patients had shorter survival than PDX engraftment-negative patients (*p* = 0.033).

It appears that oncogenic EBV, considered as potent mutation driver, accounts for relatively low mutation rate in NPC tumor [7, 10]. Consistent with previous results, we identified 34~ 99 single nucleotide variants (SNVs) for each tumor and a total 282 missense and splicing site somatic mutations in five PDX tumors originated from metastasized NPC tumors when compared with the matched patient’s peripheral blood mononuclear cells (PBMC) (Additional file 3: Figure S1 and Additional file 4: Table S2, SNV gene list, an excel file). The predominant nucleotide changes were C to T (26.2%) and G to A (20%) transitions (Additional file 3: Figure S1 lower panel).

**Fig. 1:**
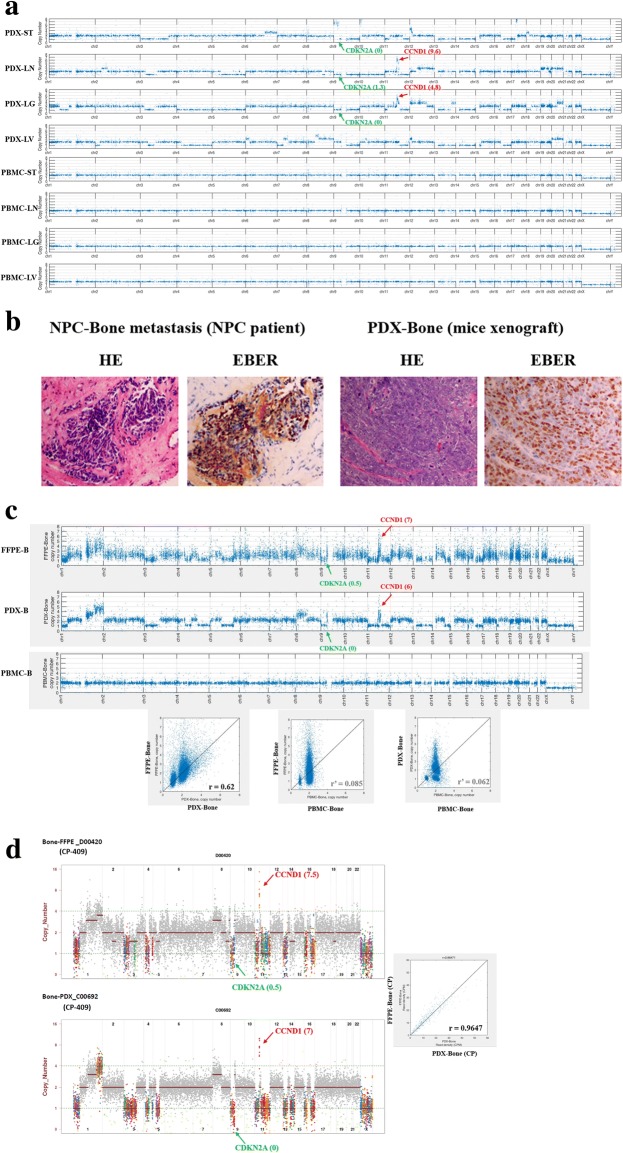
Genetic alterations in NPC-PDX tumors. (**a**) Copy number variations (CNV) of NPC-PDX tumors versus corresponding patient’s peripheral blood mononuclear cells (PBMC). Genome-wide CNV alterations in four paired PDX tumor samples (ST, LN, LG and LV). *CCND1* CNV gain (red arrow) and *CDKN2A* CNV loss (green arrow) are indicated. (**b**) HE and EB virus-encoded small RNA (EBER) staining of parental NPC tumor with bone metastasis and its derived NPC-PDX. (**c**) CNV profile comparisons of NPC FFPE-Bone and PDX-Bone based on WES and (**d**) ultra-deep sequencing of cancer panel-409 are shown (genes associated with or without copy number alteration are indicated in different colors or in grey, respectively). Observed copy number for each evaluated position is shown on the y-axis as a log 2 scale. Correlation plots with Pearson’s correlation coefficient, r, indicating similarities between two CNV profiles

In contrast to the limited somatic mutations in NPC PDX tumors, we observed genome-wide CNV affecting thousands of genes (~ 5000 genes per PDX tumor). Several chromosome regions of PDX tumors showed arm-level aberrant amplification (CNV gain) or deletion (CNV loss) as shown in Fig. 1a. Interestingly, we found that four out of five NPC-PDXs had CNV gain of *cyclin D1*, (*CCND1*, chr11-q13), and three NPC-PDXs had CNV loss of *cylin-dependent kinase inhibitor 2A*, (*CDKN2A*, chr9-p21). CCND1 protein forms a complex with cyclin-dependent kinase (CDK)4/6 and subsequently phosphorylates retinoblastoma protein leading to entry of the S phase during cell cycle [28]. CDKN2A protein, also known as p16, functions as a cell cycle inhibitor which binds to CDK4 and blocks the cyclin D1/CDK4/pRb axis by preventing cell cycle G1/S phase transition [28]. Thus, amplification of *CCND1* and deletion of *CDKN2A* are common alterations in NPC tumors [6, 10, 29–31], which may cooperatively contribute to rapid cell growth.

### PDX has high genomic fidelity to parental human NPC tumors

Both parental NPC metastatic tumors and PDX xenografts harbored EBV (with positive staining for Epstein-Barr encoding region, EBER), as shown in Fig. 1b. To determine whether the genetic compositions of PDX and original metastatic NPC tumors in patients (formalin-fixed, paraffin-embedded, FFPE) are similar, we compared their copy number (CN) profiles obtained from whole exome sequencing (with 18,070 cellular genes) and Ultra-deep sequencing cancer panel 409 (ACTOnco CP-409, containing 409 selected oncogenes and tumor suppressor genes). Pairwise comparisons revealed high correlation between the CN profiles of FFPE-Bone and PDX-Bone in both WES (Pearson correlation coefficient, *r* = 0.62; Fig. 1c) and CP-409 (*r* = 0.96; Fig. 1d). Comparable results were obtained when comparing the CN profiles of FFPE-LN and PDX-LN in CP-409 (*r* = 0.92; Additional file 5: Figure S2A and S2B). The high correlations between the CN profiles of FFPE samples and PDX tumors indicating that the PDX tumors retain the genetic composition of the parental NPC tumors.

Due to the limited somatic mutations in each NPC-PDX tumor sample, we incorporated all the SNVs identified in 282 genes as well as *CCND1* and *CDKN2A* to perform pathway analysis (Metacore). The altered cancer-related pathways identified in the NPC PDX tumors are summarized in Additional file 6: Table S3; and the most affected pathway was cell cycle. Our findings signify that amplification and/or deletion of the specific cell cycle regulators *CCND1* and *CDKN2A* are prominent abnormalities that may correlate with NPC tumorigenesis.

### Confirmation of CCND1 overexpression via WES and CNV genetic studies

**Fig. 2:**
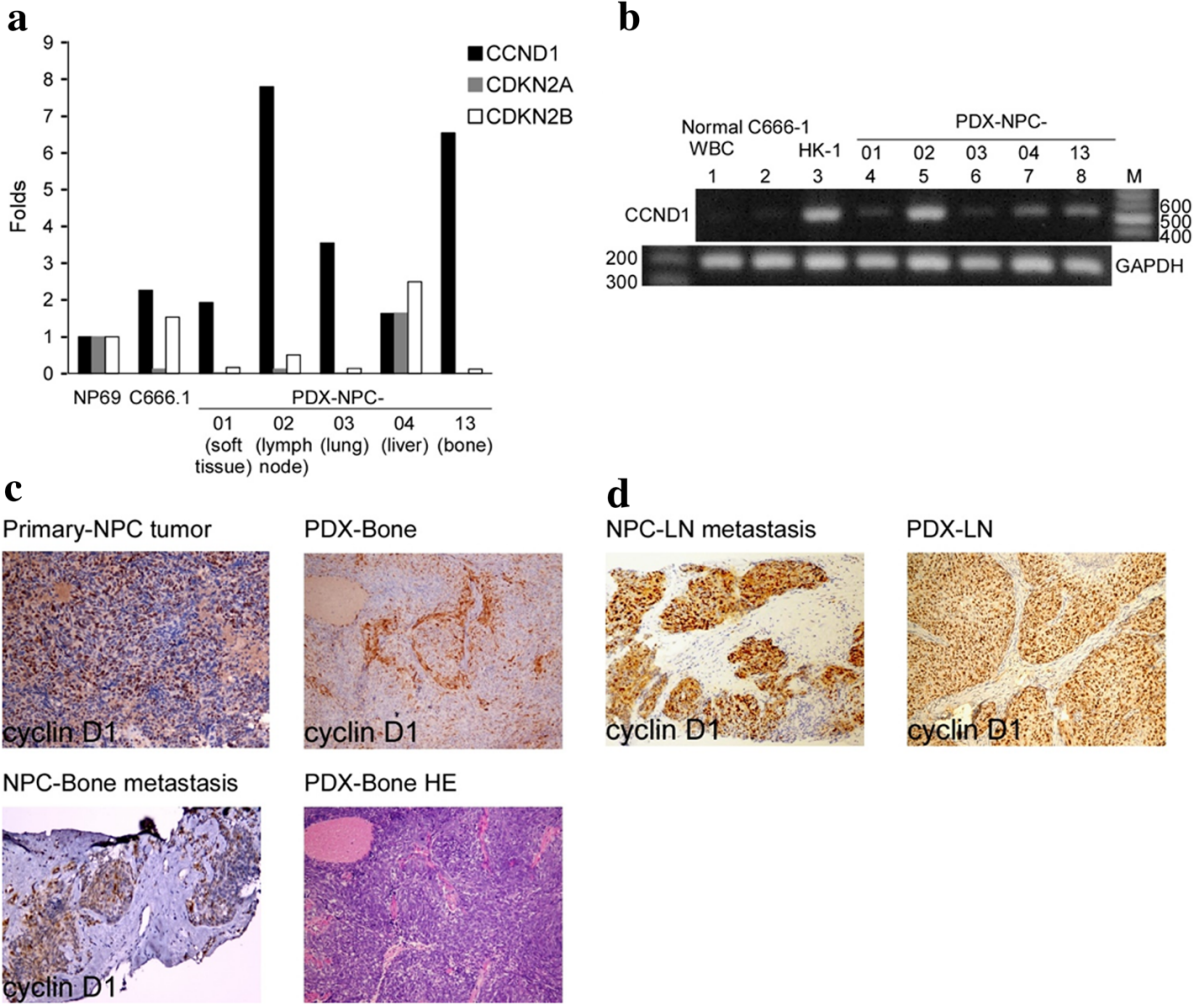
CCND1 mRNA expression and IHC staining in NPC patients and PDX tumors. (**a**) The expression fold change of candidate genes (*CCND1, CDKN2A and CDKN2B*) are indicated based on the cDNA microarray data of five PDX tissues, and C666–1 (EBV-positive NPC cells) and NP69 (immortalized normal nasopharyngeal cells, as control) cell lines. (**b**) Agarose gel electrophoresis of RT-PCR products of *CCND1* in PBMC, two NPC cell lines and five PDXs (GAPDH serves as an internal control). Cyclin D1 IHC staining in (**c**) NPC no.13 patient, with NPC primary site, NPC metastatic to bone, and PDX-Bone tumor and (**d**) NPC no.2 patient, with NPC metastatic to lymph node, and PDX-LN tumor

Microarray analyses confirmed cyclin D1 overexpression and silencing of CDKN2A and 2B in the four out of five PDXs, as shown in Fig. 2a. CCND1 mRNA overexpression (RT-PCR) was observed in NPC cell line (HK1) and five PDX tumors, (Fig. 2b). Immunohistochemical analyses further validated CCND1 protein overexpression in both parental metastatic NPC tumor and in PDX-Bone and PDX-LN, respectively (Fig. 2c and d).

### NPC-PDXs drug screening

Based on the mutations discovered by integrated genomic analyses in NPC-PDXs, we wanted to test whether cell cycle inhibitor may be used as anti-cancer drug in our NPC-PDX lines. We selected a FDA approved cell cycle inhibitor “palbociclib” (PAL, CDK4/6 inhibitor) [32] currently used in breast cancer. There are reports indicated that overexpression of epigenetics modifiers, Enhancer of Zeste homolog 2, protein methyltransferase, (EZH2) [33] and DNA methyltransferase 1 (DNMT1) [34] in NPC correlated with NPC tumorigenesis; thus, we chose EZH2 inhibitor “GSK126” and DNA methylation inhibitor “decitabine” (a nucleotide analogue of DNMT1) in our NPC-PDX drug screening model. As comparison, a conventional NPC chemodrug “gemcitabine” (GEM, a nucleotide analogue) was also included. These four drugs (GEM, GSK, DEC, and PAL) were first tested in an EBV-positive cell line, C666–1. The IC_50_ value for PAL and GSK was in the range of 10–100 μM in the C666–1 cell (Additional file 7: Figure S3A). Then we used these four drugs in PDX-C666.1 xenograft, all four drugs exerted suppressive effects on C666–1 xenograft growth when compared with DMSO control (Additional file 7: Figure S3B and S3C). Although DEC displayed toxicity to some extent, inducing body weight changes of > 20% or death in mice during treatment (Additional file 7: Figure S3D). To confirm PAL can induce growth arrest at the G_0_G_1_ phase [35], we treated C666–1 cells with 0.1, 0.5 and 1 μM PAL for 48 h and followed by flow cytometry analysis. The percentage of cells at the G_0_G_1_ phase in the PAL-treated cells increased in a dose-dependent manner when compared with that of control (DMSO) (Additional file 7: Figure S3E), indicating PAL blocks NPC cells from entering S phase.

**Fig. 3:**
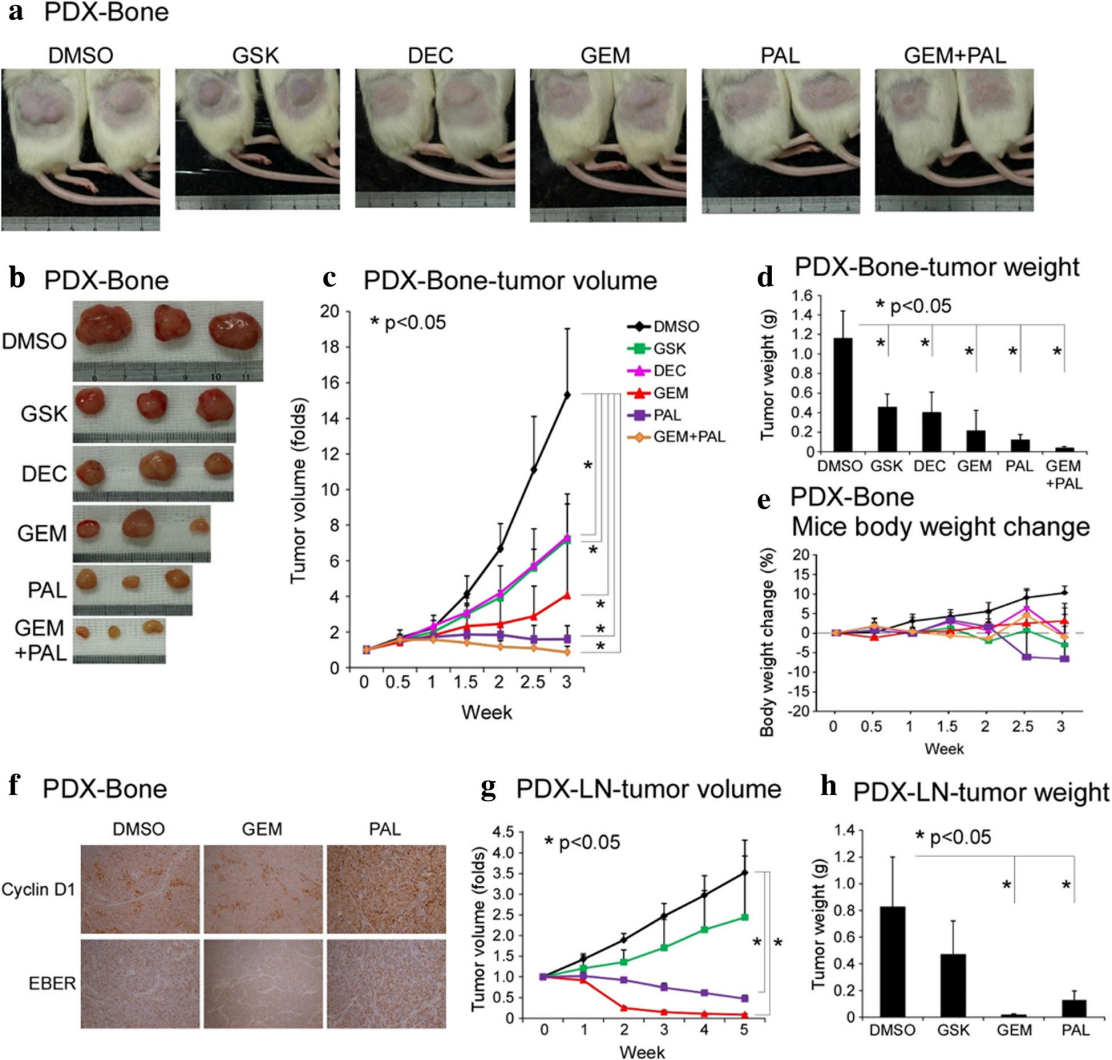
NPC-PDX drug screening. (**a**-**f**) PDX-Bone (NPC13-F4 corresponds to patient no.13 derived bone metastatic NPC tumor was transplanted in NOD/SCID mice at 4th passage); (**a**, **b**) Mice gross tumor, (**c**) tumor volume, (**d**) tumor weight, (**e**) mice body weight change, and (**f**) cyclin D1 IHC and EBER staining in control (DMSO) and GEM and PAL treatment. (**g**-**h**) PDX-LN (NPC02-F11 corresponds to patient no.2 derived lymph node metastatic NPC tumor was transplanted in NOD/SCID mice at 11th passage), (**g**) tumor volume, (**h**) tumor weight. Abbreviation: GSK, GSK126; DEC, decitabine; GEM, gemcitabine; PAL, palbociclib. (Raw data was provided in Additional file 17: Table S9 A-D)

In NPC-PDX-13-F4 (PDX-Bone passage 4) line, all four tested drugs significantly suppressed both the size (Fig. 3a-c) and gross weight of tumors in mice (Fig. 3d) relative to DMSO with tolerable body weight changes (Fig. 3e). Combination treatment with PAL and GEM induced an additive effect, compared to either GEM or PAL alone. CCND1 IHC staining after drug treatment revealed homogenous overexpression in the PAL-treated group (Fig. 3f), implying that the drug arrests the cell cycle of cancer cells at G1/S phase.

In NPC-PDX-02-F11 (PDX-LN passage 11) line, both GEM and PAL exerted significant suppressive effects on xenograft growth, but not GSK (Fig. 3g and h;). Although DEC exerted anti-tumor growth in PDX-LN, it induced toxicity and led to > 20% body loss (Additional file 8: Figure S4C). Thus, GEM and PAL had anti-tumor activity with little adverse effects in two NPC-PDX models (Additional file 8: Figure S4A-B).

### Transcriptomic analysis of NPC PDX-B with various drug treatments

To quantitatively evaluate the transcriptome profiles of poly(A) + transcripts in response to treatment with the four drugs in the NPC PDX-Bone animal model, RNA-Seq was performed (Dr. Gong Zhang and Changgong Biotech.). The mapping rate of RNA-Seq of each sample was ~ 80% (Additional file 9: Table S4). Heatmap of differentially expressed genes in drug-treated PDX-Bone mice versus control (DMSO) group was shown in Additional file 10: Figure S5. Differentially expressed genes in the drug-treated PDXs were indicated in volcano plots (Additional file 11: Figure S6A–E, left panel). The efficacy of the drugs was inversely correlated to the size of the drug-treated PDXs and positively correlated to the number of differentially expressed genes (GEM+ PAL > PAL> GEM > DEC > GSK). Differentially expressed genes were further assessed via KEGG pathway, and significant pathways indicated by the number of genes involved (size of the circle), Rich factor, and *p*-value (red to green color) (Additional file 11: Figure S6A–E, right panel). The signature-enriched pathways in GEM and PAL treatment groups were “bladder cancer and HIF-1α signaling”, and “cell cycle, p53 signaling, and ECM-receptor interaction”, respectively. Since PAL targets cell cycle-dependent kinases, it is conceivable that one of the significant pathways for this drug is cell cycle (Additional file 11: Figure S6F).

**Fig. 4:**
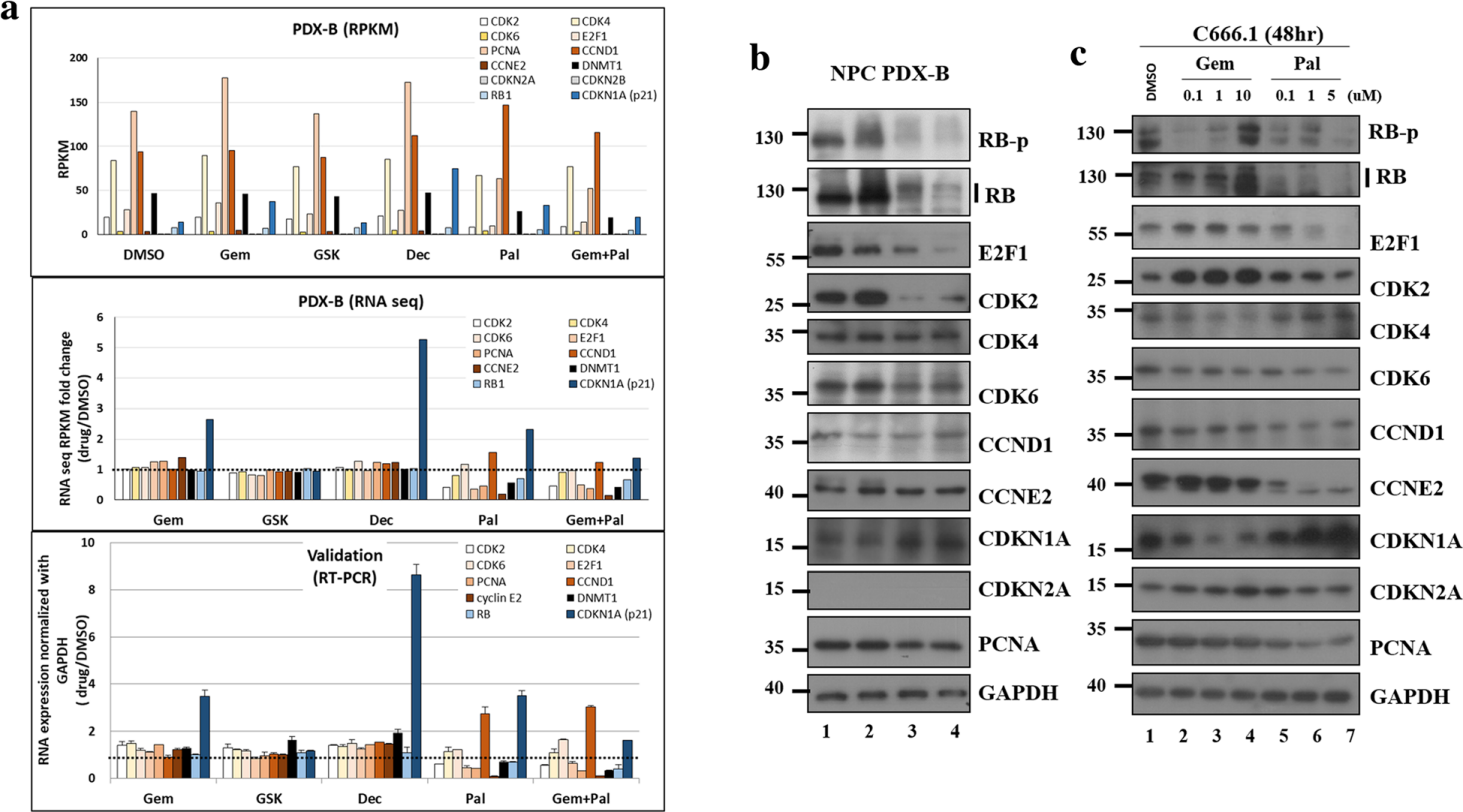
Gene expression in NPC PDX-B with different drug treatments. **a** RNA expression of nine cell cycle-related genes in NPC PDX-B exposed to five drug treatments based on original normalized (RPKM) RNA seq data (upper), fold change normalization with DMSO control (middle), RT-PCR validation fold change normalized with internal control GAPDH in DMSO (lower). **b** Western blot of the nine cell cycle-related proteins after DMSO (control), GEM, PAL and GEM+PAL treatment in NPC PDX-B tissues. **c** Western blot of the nine cell cycle-related proteins in C666–1 cells treated with different concentrations of GEM (0.1, 1, and 10 μM) and PAL (0.1, 1, and 5 μM) after 48 h

To compare the nine selected cell cycle-related genes before and after different drug treatments in PDX-Bone, normalized reads per kilobase million (RPKM; Fig. 4a, upper panel), fold change of normalized RPKM versus control (DMSO) (Fig. 4a, middle panel), and fold change of Q-RT-PCR RNA expression validation (Fig. 4a, lower panel) were determined. Since CDKN2A and 2B genes (grey bars) were deleted in NPC PDX-Bone, no RNA transcript was detected (Fig. 4a, upper panel). The gene expression fold changes following different drug treatments assayed by RNA-Seq were comparable to those assessed using Q-RT-PCR. We observed no significant fold changes in the expression of the nine genes after GSK treatment, suggesting that the EZH2 inhibitor does not target the selected cell cycle genes. Treatment with GEM, DEC and PAL induced a 3 to 5-fold increase in expression of cell arrest marker CDKN1A (p21). In addition, after PAL or GEM+PAL treatment, both RNA-Seq and Q-RT-PCR data revealed 50% ~ 80% reduction in expression of the cell cycle activators CDK2, E2F1, PCNA, CCNE2, and RB1.

Consistent with WES and RNA expression data, we observed no CDKN2A protein expression in the PDX-Bone tumor (Fig. 4b). Significant reduction in protein levels of cell cycle activators in PDX-Bone, including hyperphosphorylated RB (RB-p), total RB, E2F1 and CDK2, was evident after GEM and PAL treatment (Fig. 4b). CDK6 and PCNA protein levels were slightly decreased (Fig. 4b). On the other hand, protein expression of the cell arrest marker, CDKN1A (p21), was markedly increased (Fig. 4b). Similar results were observed in C666–1 after 48 h treatment with the two drugs (Fig. 4c). Our data collectively indicate that PAL blocks CDK activities and simultaneously reduces the protein levels of several key cell cycle activators, leading to effective suppression of PDX-Bone tumor growth in vivo.

### Correlation of CNVs of *CCND1* and *CDKN2A* with EBV DNA load in NPC patient plasma

Plasma EBV DNA load is used as a viral marker to monitor NPC tumor status; elevation of EBV DNA load in blood is usually associated with cancer recurrence/metastasis [36–39]. It is likely that EBV DNA load is related to CNV gain of *CCND1* and loss of *CDKN2A*. In this situation, PAL, may block the cell cycle effectively in NPC tumors with a *CCND1* amplification and *CDKN2A* deletion genetic background. Detection of EBV DNA load and CNVs of both *CCND1* and *CDKN2A* in liquid biopsy may have clinical value. A rapid PCR-based test was established to determine CNVs of the two cell cycle regulators in cell-free DNA. Prior to examination of plasma of NPC patients, we used genomic DNA isolated from the five PDX tumors and matched patients’ PBMC for Q-PCR amplification. Data obtained on *CCND1*, *CDKN2A* and *RAD52* (control) correlated well with CNV results determined using WES. The correlation between the two methods was high at 0.89–0.95 (Additional file 12: Table S5), suggesting that the Q-PCR assay can be effectively employed to establish the copy numbers of cellular genes.

**Fig. 5:**
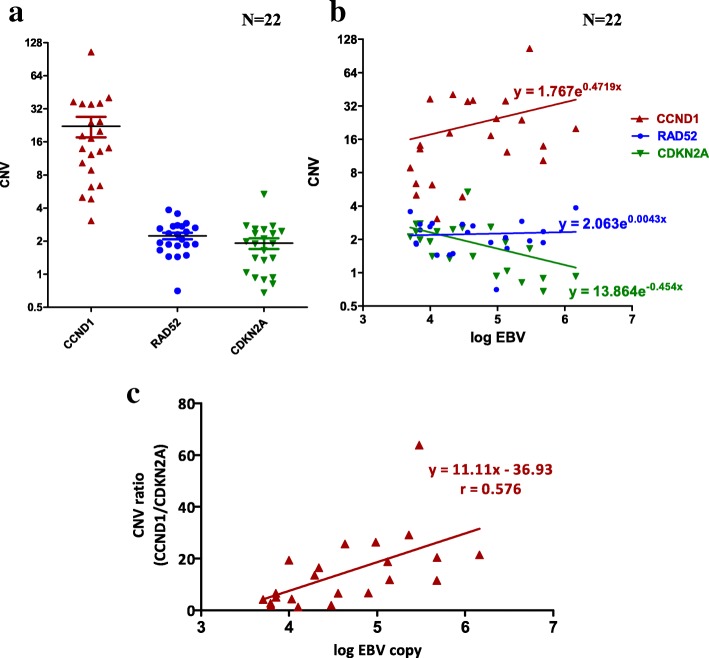
Correlation of CNV of CCND1, CDKN2A and RAD52 with high EBV copy number in 22 NPC plasma. (**a**) CNV of *CCND1*, *CDKN2A* and *RAD52* in 22 NPC plasma with high EBV DNA load (> 5000 copies/ml) based on Q-PCR results. (**b**) Correlation plot between CNV of *CCND1*, *CDKN2A* and *RAD52* and log EBV DNA load in 22 NPC plasma samples (**c**) Correlation between CNV of the *CCND1*/*CDKN2A* ratio and log EBV load in 22 NPC plasma. Pearson’s correlation coefficient, r, and equations of regression are indicated

Subsequently, we selected 22 plasma with high EBV DNA copy number (> 5000 copies/ml) collected at two different time-points from a group of 11 NPC patients and 24 plasma with low EBV DNA copy number (< 5000 copies /ml) collected from 24 NPC patients. Cell-free DNA isolated from plasma was used for Q-PCR analysis of *CCND1*, *CDKN2A* and *RAD52*. PCR results were normalized with those of healthy individual PBMC samples. For low EBV DNA load plasma (0–3000 copies/ml), weak positive correlation (*r* = 0.396) was observed between EBV DNA load and CNV of *CCND1* but no correlation between EBV DNA load and CNV of (a) *CDKN2A* (*r* = 0.082) and (b) *RAD52* (*r* = 0.25) (Additional file 13: Figure S7 and Additional file 14: Table S6B). The average CNV of the three selected cellular genes was ~ 2 (Additional file 13: Figure S7, left panel), indicating that when EBV DNA load was low in plasma (< 5000 copies/ml), CNV for *CDKN2A* and *RAD52* remained unchanged (~ 2) but CNV for *CCND1* began to increase (> 2) even in low EBV copies. For high EBV DNA load plasma (> 5000 copies/ml), average CNV for *RAD52* remained ~ 2, suggesting no correlation between EBV DNA load and CNV *RAD52* (*r* = 0.056) (Additional file 14: Table S6A). However, we observed CNV gain for *CCND1* and slight CNV loss for *CDKN2A* in plasma with high EBV DNA load (Fig. 5a). Surprisingly, the average *CCND1* CNV in the high EBV DNA load group was ~ 22. The correlation coefficients between EBV DNA load/ml (log) and CNVs of (a) *CCND1* and (b) *CDKN2A* were *r* = 0.325 (weak) and *r* = − 0.488 (moderate) (Additional file 14: Table S6A), respectively. At plasma EBV DNA loads > 100,000 copies/ml, the chance for the cell-free DNA to lose one copy of *CDKN2A* was 70% (5 out of 7, Additional file 14: Table S6A). Interestingly, we observed a better positive moderate correlation (*r* = 0.576) with EBV DNA load in plasma using the CNV ratio of *CCND1* and *CDKN2A* within the same sample instead of CNV of a single gene (Fig. 5b and Additional file 14: Table S6A). This new correlation plot showed that the risk of aberrant CNV of cell cycle regulators, *CCND1* and *CDKN2A,* in NPC tumors depends on the increased EBV DNA load in the circulation. According to the linear regression equation, y = 11.11×-36.93 (where x and y represent the log of EBV DNA load and CNV ratio of [*CCND1*/*CDKN2A*], respectively; Fig. 5b), in cases where EBV DNA load in plasma is 5000 copies/ml, the CNV ratio is ~ 4, supporting amplification of *CCND1* and/or deletion of *CDKN2A*. Thus, high EBV DNA load in the plasma is simultaneously associated with CNV gain in *CCND1* (a cell cycle accelerator), loss in *CDKN2A* (a cell cycle brake), and uncontrollable cell growth in EBV-positive NPC tumors.

### Elevated CCND1 expression as poor prognostic marker and potential treatment of PAL in NPC tumors

**Fig. 6:**
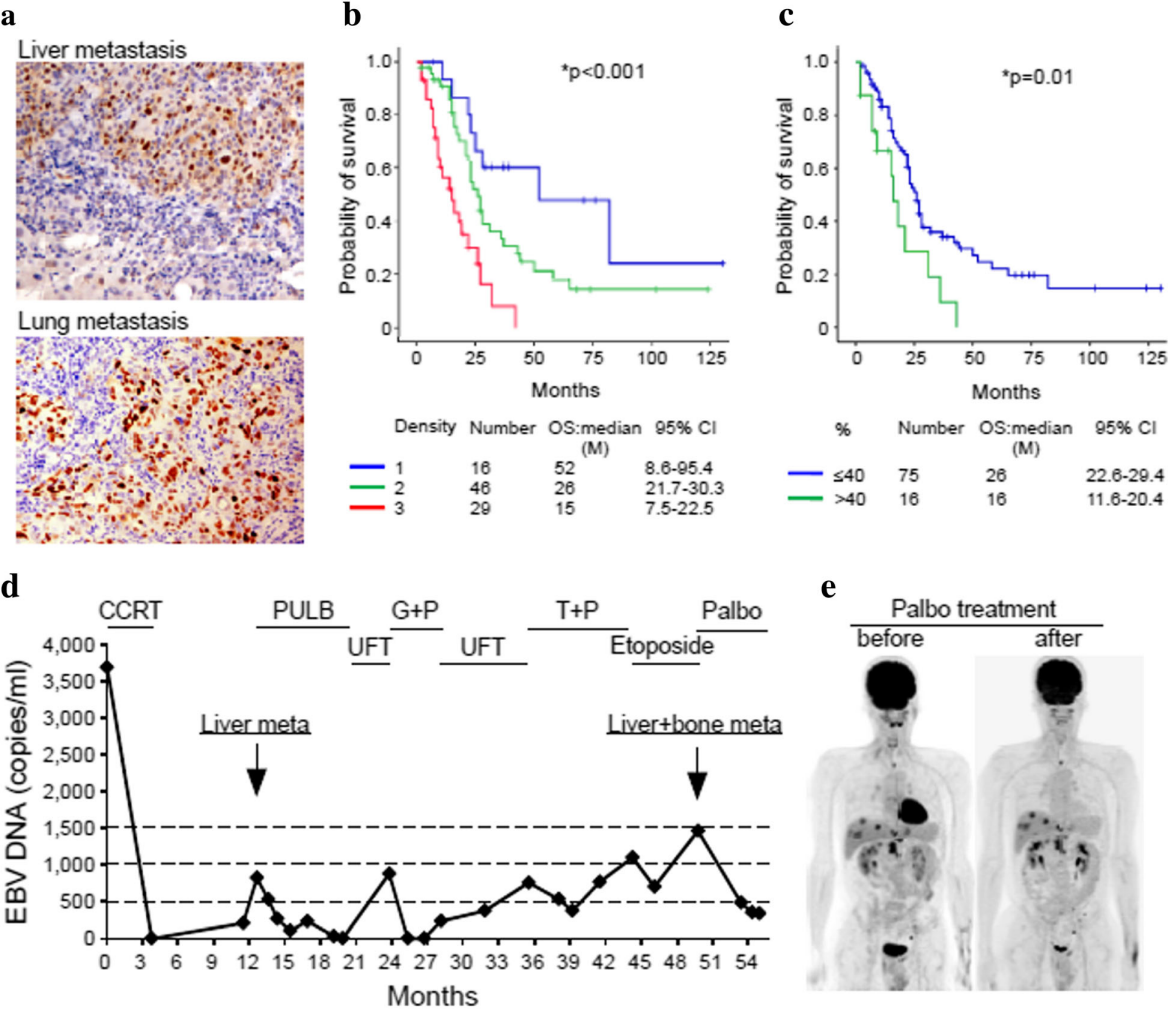
Cyclin D1 IHC in metastatic NPC clinical samples. (**a**) Cyclin D1 expression in a liver and a lung metastasized NPC patients. The Kaplan-Meier survival curves of the NPC patients classified by the (**b**) intensity and (**c**) percentage of positive cells in cyclin D1 IHC staining. A 46 year-old NPC patient with T4N2M0 (stage IVa), also a hepatitis B virus carrier, received concurrent chemoradiotherapy (CCRT) for local disease control. Liver metastasis (cyclin D1 staining, in Fig. 7a) was detected 9 months later and five different lines of palliative chemotherapy prescribed. Finally, the patient received palbociclib (PAL) as salvage treatment at Johns Hopkins Medicine, Singapore. (**d**) Plasma EBV DNA load reflecting the clinical treatment response of the patient was decreased after treatment with PAL. (**e**) Whole-body tumor scan before and after PAL treatment at 2-month intervals revealed stable disease. Abbreviations: PULB, (cisplatin, tegafur/uracil, leucovorin, and bleomycin); UFT, (tegafur/uracil); G + P, (gemcitabine + cisplatin); T + P, (docetaxel + cisplatin)

Assessment of CCND1 expression in 139 NPC FFPE samples from CGMH hospital (2002 to 2016) disclosed that only 9 samples (6.5%) had undetectable CCND1 while 130 samples (93.5%) had CCND1 overexpression. Among the CCND1 overexpressed samples, 116 samples (83.4%) showed strong CCND1 staining (2+ and 3+) (Additional file 15: Table S7). Both expression density and percentage positivity of CCND1 cells were inversely correlated with survival with statistical significance, as shown in Fig. 6b and c. Additionally, cyclin D1 was highly overexpressed in primary site tumor (87.9%) and local recurrent (93.3%) samples. Among the 91 metastatic NPC FFPE samples, 81 had matched plasma EBV DNA data. From these 81 samples whose cyclin D1 density grade correlated with mean EBV DNA load (*p* = 0.046, Additional file 16: Table S8). In addition, EBV DNA load with cutoff value of 5000 or 10,000 copies/ml was a prognostic factor for overall survival in 81 metastatic NPC samples (Additional file 13: Figure S7C and S7D). In general, the higher the EBV load, the higher the CCND1 expression and the lower the overall survival.

One NPC patient, subjected to local CCRT, developed liver, lung, and bone metastasis with high cyclin D1 expression (Fig. 6a). Although the patient received addition five lines of palliative chemotherapy, all of them failed to improve the condition. As the final attempt, the patient was further treated with two courses of PAL alone, and showed maximal grade 2 myelosuppression and decreased plasma EBV DNA load (Fig. 6d). A follow-up PET scan revealed stable disease (Fig. 6d and e). Our data suggest that PAL as salvage treatment shows anti-cancer efficacy to some extent.

## Discussion

EBV oncogenic viral genes are considered strong tumor drivers, and therefore, it is not necessary for EBV-positive tumor cells to acquire many somatic mutations. On average, ~ 100 somatic mutations were identified in each NPC tumor [10]. Previous reports have additionally suggested that EBV infection contributes to host genome instability [40–44]. Our sequencing data revealed aberrant CNV gain and loss in many chromosome regions of NPC tumors at the arm level. The roles of EBV in CNVs and genome instability in NPC thus require further evaluation. Based on WES and cancer panel CNV analyses, the global CNV profiles of PDX-B tumors shared remarkable similarities (*r* = 0.62~ 0.9) with parental tumors. Early passages of PDX line appears to preserve the unique genetic abnormalities of the primary tumor [45], consequently offering an excellent preclinical drug testing model for personalized translational medicine. Conversely, the correlations of CNVs between (i) FFPE-B tumors and peripheral blood of the same patient (PBMC-NPC-Bone) (Fig. 2c, correlation plot, middle), and (ii) PDX-B tumors and PBMC-NPC-Bone (Fig. 2c, correlation plot, right) were low, with respective correlation coefficients of r’ = 0.085 and r’ = 0.062. This finding reflects great differences in CNVs in PBMC (somatic cells) and tumor cells even in the same individual. Thus, CNV correlations may provide an index to quantitatively describe genetic variations among paired or unpaired samples based on genome-wide CNV profiles.

The p16-cyclin D1-CDK(4/6)-Rb pathway plays a critical role in governing cell cycle G1/S transition [28]. *CCND1* amplification and overexpression [46] as well as *CDKN2A* inactivation are frequent genetic alterations in many cancers [47], including NPC [31]. Recent extensive genomic studies in NPC [7, 10] and our current study have both validated that simultaneous CDKN2A copy number lost and CCND1 copy number gain are common in NPC. Thus, siRNA-mediated CCND1 knockdown has been shown to suppress proliferation in a NPC cell line [31]. Previously, seliciclib, a non-selective CDK inhibitor, was reported to exert an anti-cancer effect but induce a number of side effects in NPC patients [48]. PAL, a specific CDK 4/6 inhibitor, could cause cell cycle arrest in breast cancer cell lines with functional Rb and high CCND1 expression but low CDKN2A expression [35]. This drug has been approved to treat hormone receptor-positive, Her-2-negative advanced breast cancer [49].

The NPC PDX system developed in the current study provided a good model to validate these actionable targets. We demonstrated that the CDK 4/6 inhibitor, palbociclib, causes cell cycle arrest at the G_0_/G_1_ phase in cell lines and suppresses NPC xenograft and PDX growth. Furthermore, one heavily treated patient with liver metastases from NPC displayed stable disease with lower plasma EBV DNA load after administration of palbociclib. These results support the potential application of cell cycle inhibitors in future clinical trials for NPC.

The number of differentially expressed genes from RNA-seq data of PDX-B after drug treatment may serve as an indicator of treatment effectiveness, since the drugs can induce substantial alterations in specific gene expression patterns in PDX tumors. In general, the size of the drug-treated PDX-B tumor is inversely proportional to the number of differentially expressed genes (DMSO > GSK (248) > DEC (542) > GEM (359) > PAL (947) > GEM+PAL (1588) (Additional file 7: Figure S3A–E).

The drug screening of PDX model is indeed a time-consuming process. Based on our sequencing data, *CCND1* amplification and *CDKN2A* loss are common mutation signatures for NPC tumor; and we have demonstrated that PAL is an effective drug targeting cell cycle to suppress tumor growth in NPC PDX model with *CCND1* and *CDKN2A* CNV background. Therefore, our cell-free PCR-based CNV ratio screening test of *CCND1* and CDKN2A as well as EBV DNA load in NPC plasma may provide time-saving and valuable information to help effective monitoring NPC progression and recurrence. Once the cell-free CNV *CCND1/CDKN2A* ratio is greater than 1, then the cell cycle checkpoint is impair. Thus, the CNV *CCND1/CDKN2A* ratio may serve as a guideline to doctors whether this cell cycle-dependent kinase inhibitor, PAL treatment, has beneficial effect to the NPC patient.

Our findings indicate that plasma EBV DNA load is positively correlated with the CNV ratio of *CCND1/CDKN2A*. Hence, determination of the CNV ratio between cell-free *CCND1* and *CDKN2A* as well as EBV DNA load in NPC plasma may provide valuable information that aids in the effective monitoring of NPC progression and recurrence. In addition, the cell-free *CCND1*/*CDKN2A* ratio may signify whether patients with NPC recurrence are suitable for cell cycle-dependent kinase inhibitor PAL treatment. From experience, when the EBV DNA load in patient plasma is greater than 5000 copies/ml, the condition of the patient gradually deteriorates and thus more active medical measures should be employed. The cut-off value for the CNV *CCND1*/*CDKN2A* ratio was set to 4, which corresponds to EBV DNA load of ~ 5000 copies/ml in plasma. We propose that this CNV ratio, in combination with EBV DNA load in plasma, may be effectively utilized as a guideline for individualizing treatments for NPC patients.

## Conclusions

We have successfully established five novel NPC patient derived xenograft tumor mice model for the study of genetic alterations and possible treatment in NPC tumor. By integrating genomic and transcriptomic studies of NPC PDX model, we were able to discover copy number of CCND1 and CDKN2A are the potential drug target of CDK inhibitor, palbociclib, to suppress tumor growth. We also developed a PCR-based CNV test to determine whether a NPC patient may be suitable for palbociclib treatment.

## Additional files

Additional file 1: Supplementary Materials and Methods. (DOCX 16 kb)

Additional file 2: **Table S1.** Characteristics of total 17 metastatic NPC patients and the final 5 NPC-PDX lines (grey). (PDF 401 kb)

Additional file 3: **Figure S1.** Single nucleotide variations (SNV) of five metastatic NPC-PDX tumors. Somatic mutations (including non-synonymous missense and splice site mutations) of the five NPC-PDX tumors identified from sequencing data are listed (upper panel). Each bar represents the number of base substitutions. For the 282 SNVs, the percentage of each base substitution is indicated (lower panel). (PDF 402 kb)

Additional file 4: **Table S2.** Summary of 282 SNV mutations in 5 NPC-PDX tumors. (XLSX 32 kb)

Additional file 5: **Figure S2.** CNV profile comparison between CP-409 FFPE-LN and PDX-LN. CNV profiles of NPC (A) FFPE-LN and (B) PDX-LN based on ultra-deep sequencing of CP-409. Observed copy number for each evaluated position is shown on the y-axis as a log 2 scale. Genes associated with or without copy number alteration are indicated in different colors or in grey, respectively. Correlation plots with Pearson’s correlation coefficient, r, is indicated. (PDF 467 kb)

Additional file 6: **Table S3.** The summary of the cancer-related somatic mutations and *CCND1* CNV gain and *CDKN2A* CNV loss of the 5 NPC-PDX tumors. (PDF 314 kb)

Additional file 7: **Figure S3.** C666.1 cells and PDX-C666.1 xenograft drug screening. Drug sensitivity tests in (A) C666.1 cells and (B-D) PDX-C666.1 xenograft. The changes in PDX-C666.1 (B) tumor volume, (C) tumor weight (g), and (D) mice body weight are indicated. Abbreviation, GSK, GSK126; DEC, decitabine; GEM, gemcitabine; PAL, palbociclib. (E) Flow cytometry analysis of C666.1 cells in the presence of PAL (0, 0.1, 0.5 and 1 μM). (PDF 485 kb)

Additional file 8: **Figure S4.** Drug screening in PDX-LN (NPC02F12). PDX-LN (A) tumor volume; (B) tumor weight; and (C) mice body weight change in the presence of DMSO (control), DEC (reduced dose) and PAL. (PDF 395 kb)

Additional file 9: **Table S4.** RNA-seq mapping statistics of PDX-Bone in the presence of five drug treatments. (PDF 407 kb)

Additional file 10: **Figure S5.** Heatmap of RNA-seq data of PDX-B and with 5 drug treatments. RNA expression profiles of PDX-B (DMSO, control) and treated with 1. GEM, 2. GSK126, 3. DEC, 4. PAL, 5. GEM+PAL. The color scale (red, yellow and blue) indicates the expression level (log10 RPKM) from high to low. (PDF 375 kb)

Additional file 11: **Figure S6.** Differentially expressed genes of PDX-Bone exposed to five different drugs. Volcano plots (left panel) generated by analysis of differential gene expression based on RNA sequencing data on PDX-Bone with five drug treatments: (A) GEM, (B) GSK126, (C) DEC, (D) PAL, and (E) GEM + PAL. The x-axis represents the log_2_ base fold change of PDX-B treated with different drugs and y-axis represents the *p*-value (−log10) for differential gene expression. Other genes that passed quality control are presented as gray dots. Up-regulated (red dots) and downregulated (green dots) genes are indicated. Enriched KEGG cellular pathways (right panel) are presented as circles according to scores from enrichment *p*-value (−log10) (y-axis) and topology analysis (pathway enrichment factor, x-axis). The circle color scale (red to green) indicates the significance of the pathway. The size of the circle represents the number of genes involved in the pathway. (F) The KEGG cell cycle pathway. Green boxes represent downregulated genes in the cell cycle pathway of PDX-B treated with PAL. (PDF 961 kb)

Additional file 12: **Table S5.** Correlation between CNV detected by WES and Q-PCR. (PDF 298 kb)

Additional file 13: **Figure S7.** Correlation between CNV of cellular genes and low EBV copy number in NPC plasma. (A) The CNV of CCND1, CDKN2A and RAD52 in 24 NPC plasma with low EBV DNA load (< 5000 copies/ml) based on the Q-PCR results. (B) Correlation plot between the CNV of CCND1, CDKN2A and RAD52 versus log EBV DNA load (low copy) in 24 NPC plasma. Pearson’s correlation coefficient, r, and exponential regression trend lines are indicated. (C). Overall survival in 81 metastatic NPC patients with EBV copy cut off (5000 copies/ml and 10,000 copies/ml) in plasma (2002–2016). Clinical characteristics of metastatic NPC patients with FFPE tissue cyclin D1 immnunohistochemical staining (2002–2016) was summarized in Additional file 16: Table S8. (PDF 381 kb)

Additional file 14: **Table S6.** CNV of CCND1, p16 and RAD52 in the 11 NPC patients’ plasma with (A) high and (B) low EBV copy number. (PDF 783 kb)

Additional file 15: **Table S7.** Immnunohistochemical (IHC) staining of cyclin D1 in 139 NPC tissues from year 2002 to 2016. (PDF 415 kb)

Additional file 16: **Table S8.** Clinical characteristics of metastatic NPC patients with FFPE tissue cyclin D1 immnunohistochemical staining (2002–2016). (PDF 364 kb)

Additional file 17: **Table S9.** PDX tumor growth during drug treatment (A) PDX-Bone tumor volume (mm^3^) and tumor weight (g), and (B) PDX-Bone mice body weight (g) in drug screening; (C) PDX-LN tumor volume (mm^3^) and tumor weight (g), and (D) PDX-LN mice body weight (g) in drug screening. (PDF 241 kb)

## Abbreviations

CCND1: Cyclin D1
CCRT: Chemoradiotherapy
CDK: cyclin-Dependent kinase
CDKN2A: Cyclin-dependent kinase Inhibitor 2A
CDKN2B: Cyclin-dependent kinase Inhibitor 2B
CNV: Copy number variation
DEC: Decitabine
DNMT1: DNA methyltransferase 1
EBV: Epstein Barr virus
EZH2: Enhancer of Zeste homolog 2
FFPE: Formalin-fixed, paraffin-embedded
G + P: (Gemcitabine + cisplatin)
GEM: Gemcitabine
GSK: GSK126
IHC: Immunohistochemistry
NPC: Nasopharyngeal carcinoma
PAL: Palbociclib
PBMC: Peripheral blood mononuclear cells
PDX: Patient derived xenograft
PDX-B: PDX-Bone
PDX-LG: PDX-lung
PDX-LN: PDX-lymph node
PDX-LV: PDX-liver
PDX-ST: PDX-soft tissue
PULB: (Cisplatin, tegafur/uracil, leucovorin, and bleomycin)
RPKM: Reads per kilobase per million mapped reads
SNV: Single nucleotide variant
T + P: (Docetaxel + cisplatin)
UFT: (Tegafur/uracil)
WES: Whole-exome sequencing
