# Supplementary material for: Integrated genomic analyses in PDX model reveal a cyclin-dependent kinase inhibitor Palbociclib as a novel candidate drug for nasopharyngeal carcinoma
Source: J Exp Clin Cancer Res. 2018 Sep 20;37:233. doi: 10.1186/s13046-018-0873-5 (PMC6149192; doi:10.1186/s13046-018-0873-5)
Supplement: Supplementary file 1 — Supplementary Materials and Methods. (DOCX 16 kb) [file 13046_2018_873_MOESM1_ESM.docx]

**Integrated genomic analyses in PDX model reveal a cyclin-dependent kinase inhibitor Palbociclib as a novel candidate drug for nasopharyngeal carcinoma**

**Additional file 1: supplementary materials and methods**

**Materials and Methods**

**Cell cycle analysis**

Cells (1 x 10^6^) were seeded on a 10 cm dish in complete medium with 10% FBS for 24 h. The medium was replaced with basal medium devoid of FBS for a further 24 h. Next, cells were treated with complete medium containing 10% FBS plus 0-1 μM palbociclib for 24 h, trypsinized, and fixed in 70% ethanol at 4^o^C for 30 min. Fixed cells were washed with PBS, treated with 100 μg/mL RNase and stained with 50 μg/mL Propidium Iodide. Following staining, cells were washed with PBS and analyzed using the Navios (TM) Flow Cytometer (Beckman Coulter). Data were evaluated using Kaluza Flow Cytometry Analysis Software.

**Drug sensitivity tests in the PDX model**

After tumors had been sub-implanted in NOD/SCID mice and xenografts had reached a volume of 50~150 mm^3^, animals were randomized (3–5 mice with tumors on the flank per group) and administered with various drugs, intraperitoneal injection: gemcitabine, GSK-126, decitabine; and oral lavage: palbociclib. The following dose schedules were used: gemcitabine (2 mg/kg, 5 times/wk), GSK-126 (2.5 mg/kg, 5 times/wk), decitabine (2.5 mg/kg, 3 times/wk), and palbociclib (150 mg/kg, 5 times/wk). Gemcitabine, GSK-126, and decitabine were dissolved in DMSO and palbociclib was dissolved in distilled water. The EBV-positive cell line, C666-1, mice xenograft served as the control. Tumor dimensions were measured twice a week with calipers, and tumor volume was calculated with the formula, tumor volume (mm^3^) = a (length, mm) x b^2^ (width, mm) x 0.5. Tumors were harvested for further analysis. Three to five mice for each group were used. Mice were sacrificed ~1 month after chemical injection or earlier if tumors reached a size greater than 2000 mm^3^, body weight loss exceeded 20%, mice were unable to maintain their normal food and water intake for 3 days, had micturition or defecation difficulties, or other conditions that would violate humane treatment regulations. Final tumor volumes were compared using two-tailed ANOVA adjusted for multiple comparisons.
