# Supplementary material for: Integrated genomic analyses in PDX model reveal a cyclin-dependent kinase inhibitor Palbociclib as a novel candidate drug for nasopharyngeal carcinoma
Source: J Exp Clin Cancer Res. 2018 Sep 20;37:233. doi: 10.1186/s13046-018-0873-5 (PMC6149192; doi:10.1186/s13046-018-0873-5)
Supplement: Supplementary file 2 — Table S1. Characteristics of total 17 metastatic NPC patients and the final 5 NPC-PDX lines (grey). (PDF 401 kb) [file 13046_2018_873_MOESM2_ESM.pdf]

Table S1. Characteristics of total 17 metastatic NPC patients and the final 5 NPC-PDX lines (grey)

Patient No.	Age (y/o)	Gender	Status at biopsy/operation			Previous treatment	PDX engraft	Overall survival (months)	PDX Abb.
			Tissue source	Biopsy(B)/ Excision(E)	EBV DNA load (copies/ml)				
1	42	Male	Soft tissue	E	4,525	No	Yes	13	PDX-ST (01)
2	42	Male	Neck LN	B	326,000	CCRT	Yes	14	PDX-LN (02)
3	64	Male	Lung	B	87	CCRT	Yes	15	PDX-LG (03)
4	52	Male	Liver	E	3,060	CCRT	Yes	7	PDX-Lv (04)
6	63	Female	Lung	E	680	CCRT	No	28	
7	58	Male	Lung	E	704	CCRT	No	20	
8	48	Male	Liver	B	29,020	CCRT	No	18	
9	45	Male	Lung	E	4,526	CCRT	No	26	
10	28	Male	Lung	E	11,550	CCRT	No	17	
12	52	Male	Neck LN	B	1,800,000	CCRT	No	20	
13	52	Male	Bone	B	>2,000,000	CT	Yes	5	PDX-Bone (13)
16	15	Male	Neck LN	B	4,891	No	No	14	
17	46	Male	Neck LN	B	23,348	No	No	14	
18	52	Male	Axillary LN	B	721	CCRT	No	6	
19	58	Male	Lung	E	704	CCRT	No	10	
20	54	Male	Neck LN	B	39,796	CCRT	No	12	
21	52	Male	Liver	B	3,361	No	No	14	

1. CCRT: concurrent chemoradiotherapy; LN: lymph node; CT: chemotherapy
2. PDX engraft rate: 5/17 (29.4%)
3. Overall survival was defined from taking tissue for PDX to death; PDX engraft positive: negative, p=0.033
4. Biopsy: 10/17 (58.8%)
5. Male: 16/17 (94.1%)
