# Supplementary figures and images for: Integrated genomic analyses in PDX model reveal a cyclin-dependent kinase inhibitor Palbociclib as a novel candidate drug for nasopharyngeal carcinoma
Source: J Exp Clin Cancer Res. 2018 Sep 20;37:233. doi: 10.1186/s13046-018-0873-5 (PMC6149192; doi:10.1186/s13046-018-0873-5)

Fig. S1

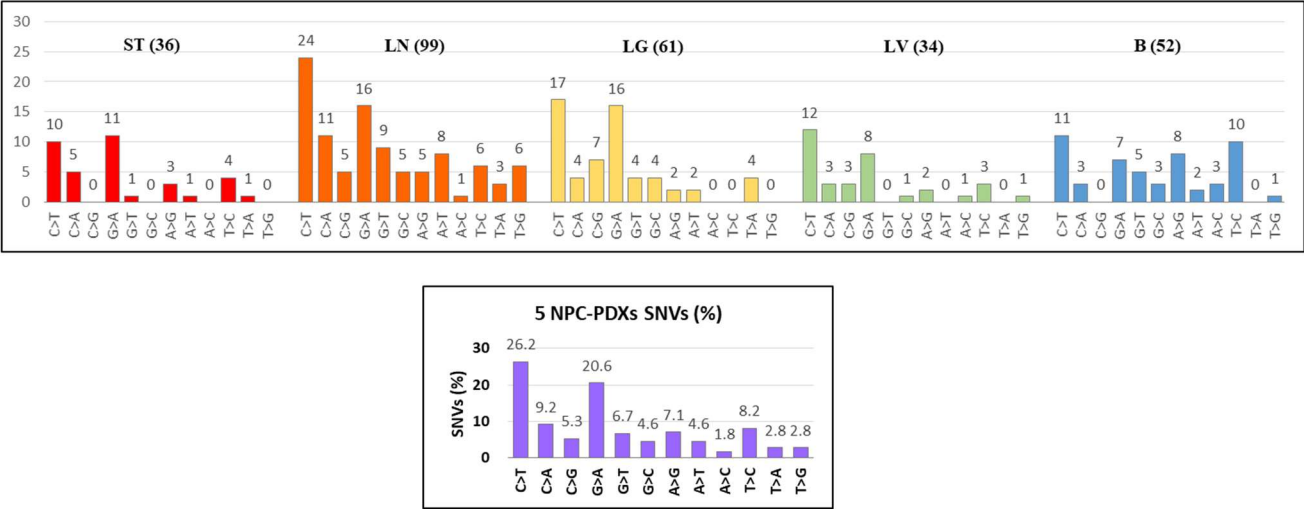

Fig. S1. Single nucleotide variations (SNVs) of the 5 metastatic NPC-PDXs.

Supplement: Supplementary file 3 — Figure S1. Single nucleotide variations (SNV) of five metastatic NPC-PDX tumors. Somatic mutations (including non-synonymous missense and splice site mutations) of the five NPC-PDX tumors identified from sequencing data are listed (upper panel). Each bar represents the number of base substitutions. For the 282 SNVs, the percentage of each base substitution is indicated (lower panel). (PDF 402 kb) [file 13046_2018_873_MOESM3_ESM.pdf]

Fig. S2

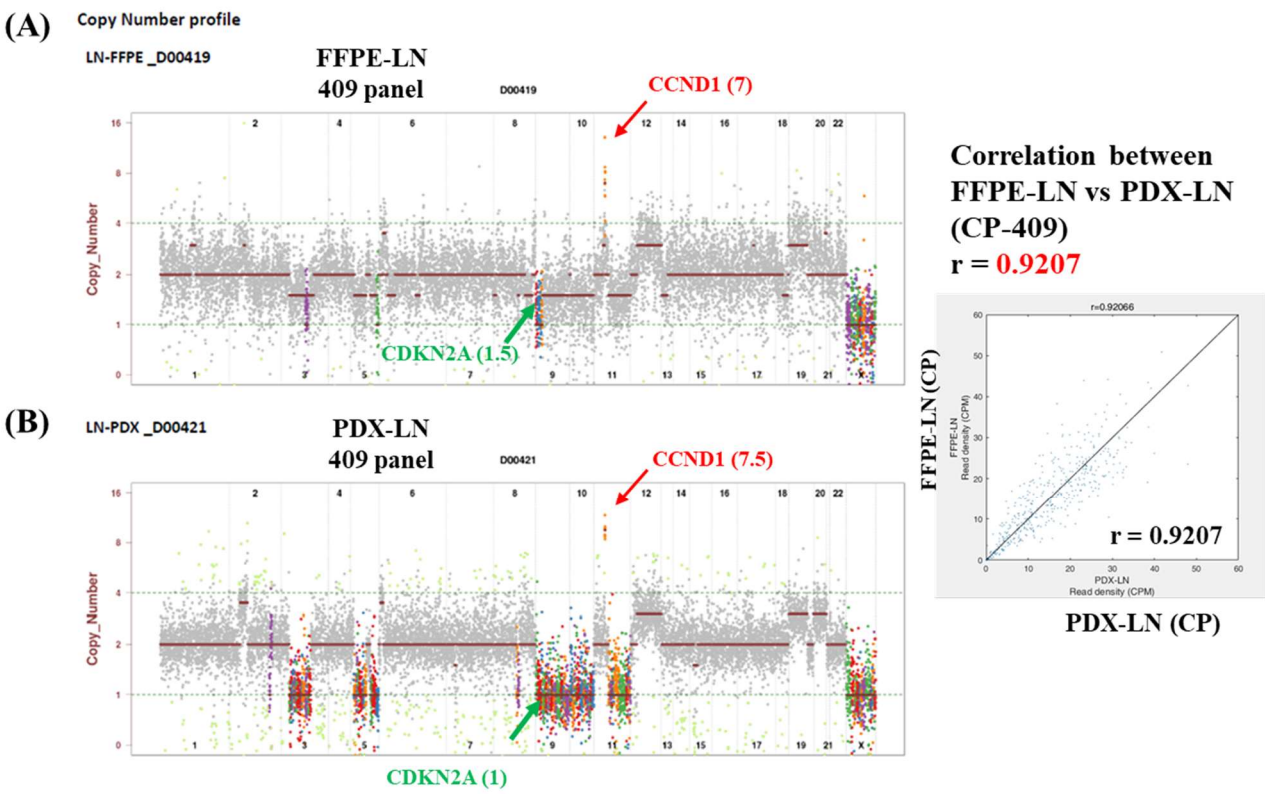

Fig. S2. CNV profile comparison between CP-409 FFPE-LN and PDX-LN.

Supplement: Supplementary file 5 — Figure S2. CNV profile comparison between CP-409 FFPE-LN and PDX-LN. CNV profiles of NPC (A) FFPE-LN and (B) PDX-LN based on ultra-deep sequencing of CP-409. Observed copy number for each evaluated position is shown on the y-axis as a log 2 scale. Genes associated with or without copy number alteration are indicated in different colors or in grey, respectively. Correlation plots with Pearson’s correlation coefficient, r, is indicated. (PDF 467 kb) [file 13046_2018_873_MOESM5_ESM.pdf]

**Fig. S3**

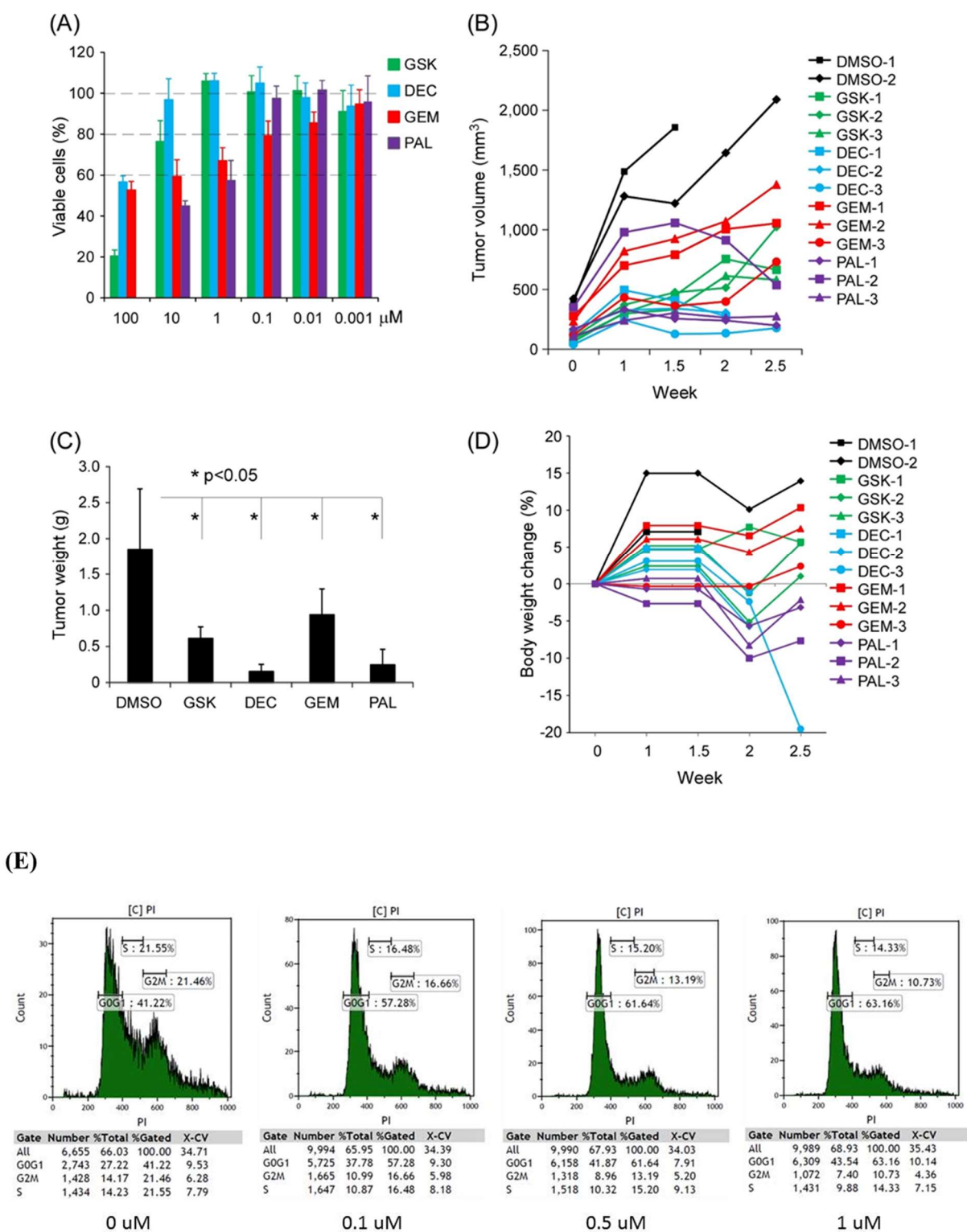

Flow cytometry analysis of C666.1 in the presence of DMSO (control) and PAL (0.1, 0.5, 1 μM)

Supplement: Supplementary file 7 — Figure S3. C666.1 cells and PDX-C666.1 xenograft drug screening. Drug sensitivity tests in (A) C666.1 cells and (B-D) PDX-C666.1 xenograft. The changes in PDX-C666.1 (B) tumor volume, (C) tumor weight (g), and (D) mice body weight are indicated. Abbreviation, GSK, GSK126; DEC, decitabine; GEM, gemcitabine; PAL, palbociclib. (E) Flow cytometry analysis of C666.1 cells in the presence of PAL (0, 0.1, 0.5 and 1 μM). (PDF 485 kb) [file 13046_2018_873_MOESM7_ESM.pdf]

**Fig. S4**

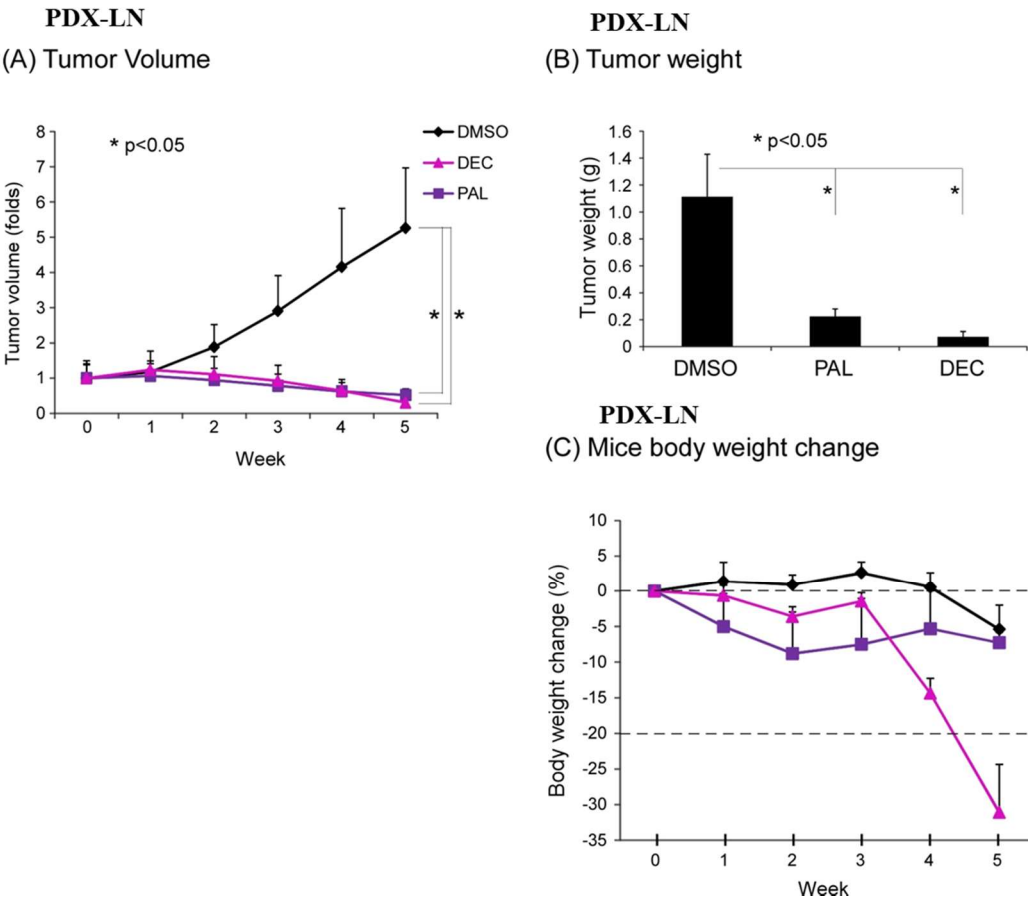

**Fig. S4. Drug screening in PDX-LN (NPC-LN-02-F12).**

Supplement: Supplementary file 8 — Figure S4. Drug screening in PDX-LN (NPC02F12). PDX-LN (A) tumor volume; (B) tumor weight; and (C) mice body weight change in the presence of DMSO (control), DEC (reduced dose) and PAL. (PDF 395 kb) [file 13046_2018_873_MOESM8_ESM.pdf]

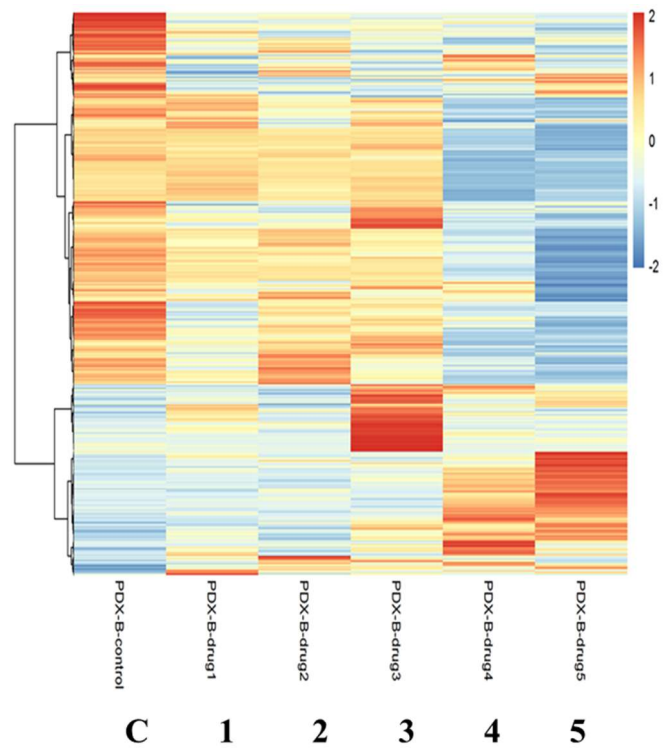

**Fig. S5.** Heatmap of RNA-seq data of PDX-B and with 5 drug treatments.

Supplement: Supplementary file 10 — Figure S5. Heatmap of RNA-seq data of PDX-B and with 5 drug treatments. RNA expression profiles of PDX-B (DMSO, control) and treated with 1. GEM, 2. GSK126, 3. DEC, 4. PAL, 5. GEM+PAL. The color scale (red, yellow and blue) indicates the expression level (log10 RPKM) from high to low. (PDF 375 kb) [file 13046_2018_873_MOESM10_ESM.pdf]

Fig. S7

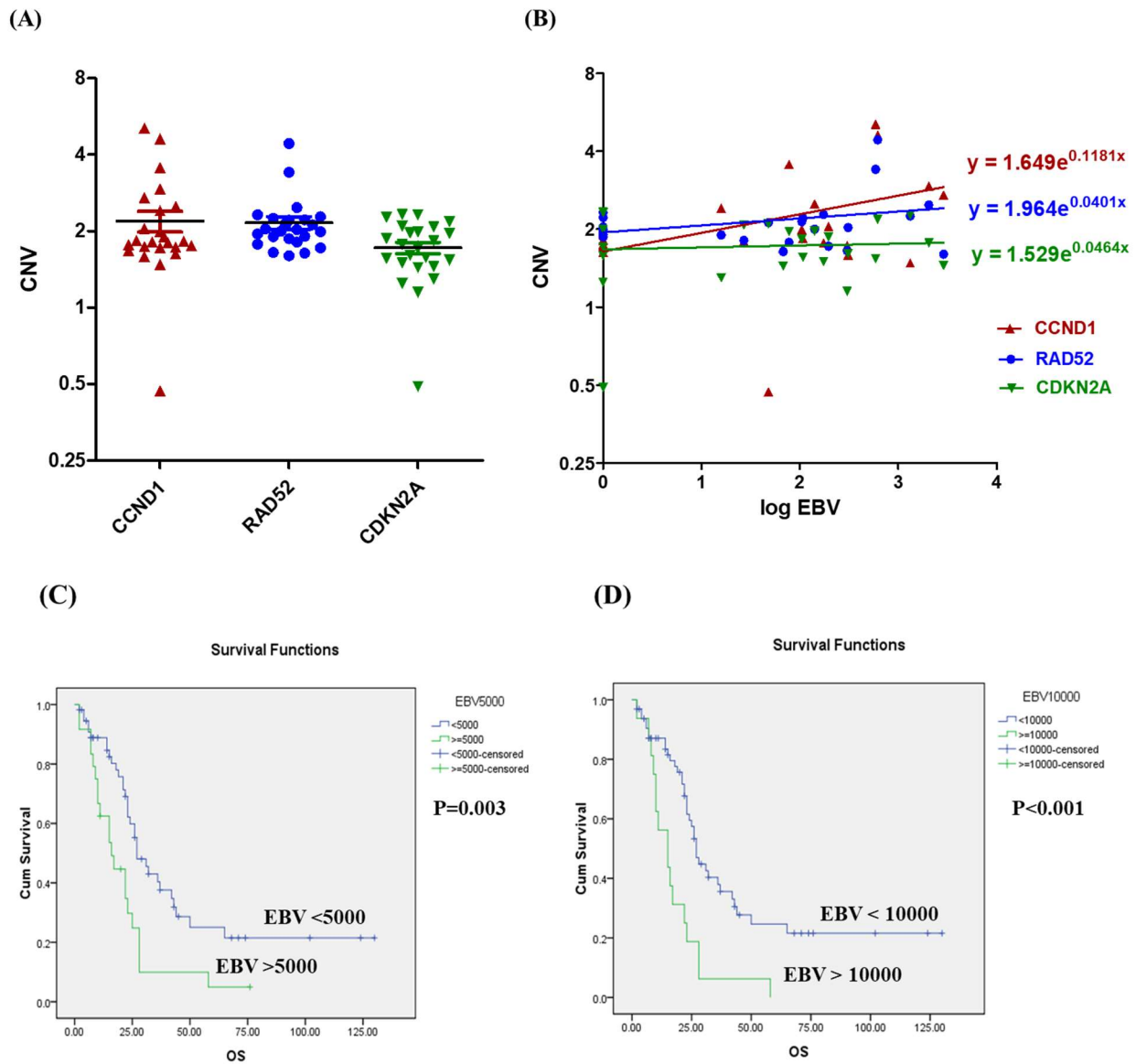

Fig. S7. Correlation between CNV of cellular genes and low EBV copy number in NPC plasma.

Supplement: Supplementary file 13 — Figure S7. Correlation between CNV of cellular genes and low EBV copy number in NPC plasma. (A) The CNV of CCND1, CDKN2A and RAD52 in 24 NPC plasma with low EBV DNA load (< 5000 copies/ml) based on the Q-PCR results. (B) Correlation plot between the CNV of CCND1, CDKN2A and RAD52 versus log EBV DNA load (low copy) in 24 NPC plasma. Pearson’s correlation coefficient, r, and exponential regression trend lines are indicated. (C). Overall survival in 81 metastatic NPC patients with EBV copy cut off (5000 copies/ml and 10,000 copies/ml) in plasma (2002–2016). Clinical characteristics of metastatic NPC patients with FFPE tissue cyclin D1 immnunohistochemical staining (2002–2016) was summarized in Additional file 16: Table S8. (PDF 381 kb) [file 13046_2018_873_MOESM13_ESM.pdf]
