# Supplementary material for: Integrated genomic analyses in PDX model reveal a cyclin-dependent kinase inhibitor Palbociclib as a novel candidate drug for nasopharyngeal carcinoma
Source: J Exp Clin Cancer Res. 2018 Sep 20;37:233. doi: 10.1186/s13046-018-0873-5 (PMC6149192; doi:10.1186/s13046-018-0873-5)
Supplement: Supplementary file 6 — Table S3. The summary of the cancer-related somatic mutations and CCND1 CNV gain and CDKN2A CNV loss of the 5 NPC-PDX tumors. (PDF 314 kb) [file 13046_2018_873_MOESM6_ESM.pdf]

Table S3. The summary of the cancer-related somatic mutations and *CCND1* CNV gain and *CDKN2A* CNV loss of the 5 NPC-PDX tumors.

Pathway	Gene	PDX-ST 01	PDX-LN 02	PDX-LG 03	PDX-LV 04	PDX-B 13
Cell cycle	*CCND1*		9.6	4.8, missense		6
	CDKN2A	0	1.3	0		0
	ANAPC7					
	CCNB1					
	CCNB3					
	CDC7					
Tumor Suppressor Genes	*AIM1*					
	DLEU7					
	ING1					
	TIMP3		splice			
Immunity	*HLA-A*				stop	
	HLA-DQA2					
	HLA-DRB5					
	IL1R2					
	TLR3					
DNA repair	*TP53*				stop	
	BRIP1					
Epigenetic modifiers	*TET3*					
	KDM2A					
Cytoskeleton	*ARHGEF12*	stop				
	ARHGEF3					
Drug Resistance	*ABCG1*					

	CNV gain
	CNV loss
	missense
splice	splice site
stop	stop gain
