# Supplementary material for: Integrated genomic analyses in PDX model reveal a cyclin-dependent kinase inhibitor Palbociclib as a novel candidate drug for nasopharyngeal carcinoma
Source: J Exp Clin Cancer Res. 2018 Sep 20;37:233. doi: 10.1186/s13046-018-0873-5 (PMC6149192; doi:10.1186/s13046-018-0873-5)
Supplement: Supplementary file 9 — Table S4. RNA-seq mapping statistics of PDX-Bone in the presence of five drug treatments. (PDF 407 kb) [file 13046_2018_873_MOESM9_ESM.pdf]

Table S4. RNA-seq mapping statistics of PDX-Bone in the presence of five drug treatments

Samples	Total Reads	Mapped Reads	Mapping Rate	has_mRNA_ mapped Reads (hg19)	mmu_mRNA_ mapped Reads (mm10)	lncRNA_ mapped Reads	Virus _ mapped Reads
C. PDX-B Con	58,401,590	46,254,346	79.20%	42,263,187	2,903,561	1,048,497	39,101
1. PDX-B GEM	49,892,923	40,809,157	81.79%	36,141,204	3,880,058	747,034	40,861
2. PDX-B GSK	52,431,848	41,927,970	79.97%	38,660,867	2,285,473	938,664	42,966
3. PDX-B DEC	43,372,857	35,266,107	81.31%	31,677,112	2,860,299	696,647	32,049
4. PDX-B PAL	28,730,565	23,583,377	82.08%	20,456,801	2,659,627	429,511	37,438
5. PDX-B GEM+PAL	32,784,657	27,282,576	83.22%	23,489,568	3,196,872	540,098	56,038

The mapping rate of RNA-Seq of each sample was ~80%. EBV transcripts comprised 0.1–0.2% of the total mapped transcripts
