# Supplementary material for: Integrated genomic analyses in PDX model reveal a cyclin-dependent kinase inhibitor Palbociclib as a novel candidate drug for nasopharyngeal carcinoma
Source: J Exp Clin Cancer Res. 2018 Sep 20;37:233. doi: 10.1186/s13046-018-0873-5 (PMC6149192; doi:10.1186/s13046-018-0873-5)
Supplement: Supplementary file 11 — Figure S6. Differentially expressed genes of PDX-Bone exposed to five different drugs. Volcano plots (left panel) generated by analysis of differential gene expression based on RNA sequencing data on PDX-Bone with five drug treatments: (A) GEM, (B) GSK126, (C) DEC, (D) PAL, and (E) GEM + PAL. The x-axis represents the log2 base fold change of PDX-B treated with different drugs and y-axis represents the p-value (−log10) for differential gene expression. Other genes that passed quality control are presented as gray dots. Up-regulated (red dots) and downregulated (green dots) genes are indicated. Enriched KEGG cellular pathways (right panel) are presented as circles according to scores from enrichment p-value (−log10) (y-axis) and topology analysis (pathway enrichment factor, x-axis). The circle color scale (red to green) indicates the significance of the pathway. The size of the circle represents the number of genes involved in the pathway. (F) The KEGG cell cycle pathway. Green boxes represent downregulated genes in the cell cycle pathway of PDX-B treated with PAL. (PDF 961 kb) [file 13046_2018_873_MOESM11_ESM.pdf]

Fig. S6A

359

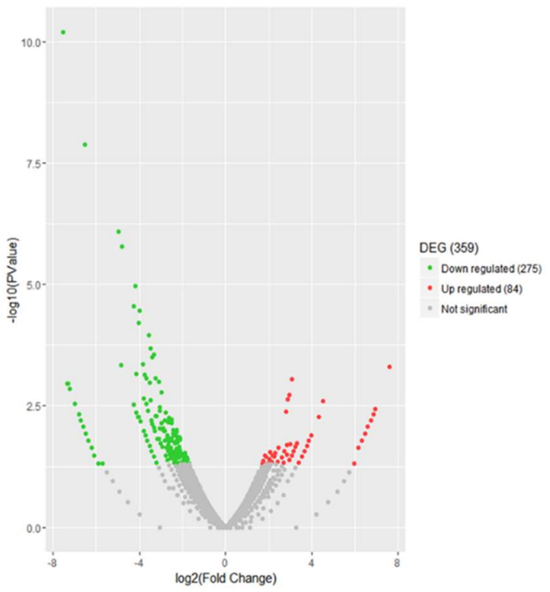

GEM

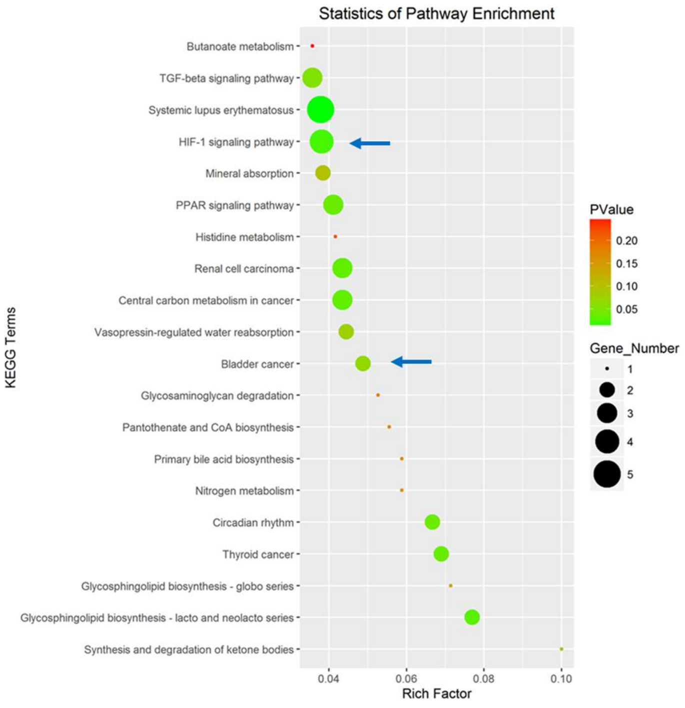

GEM

Fig. S6B

248

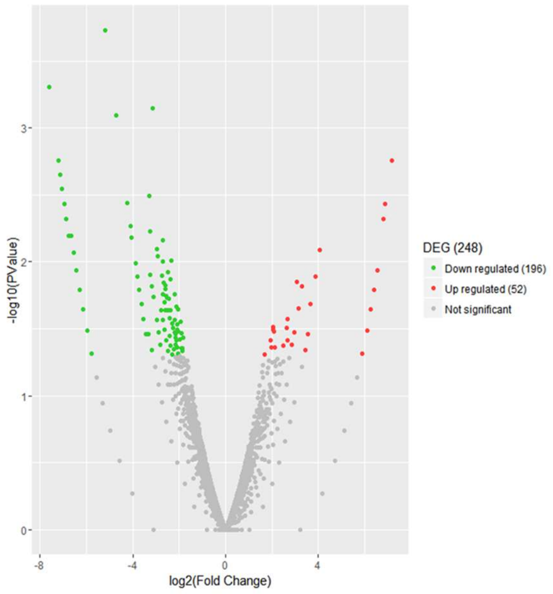

GSK

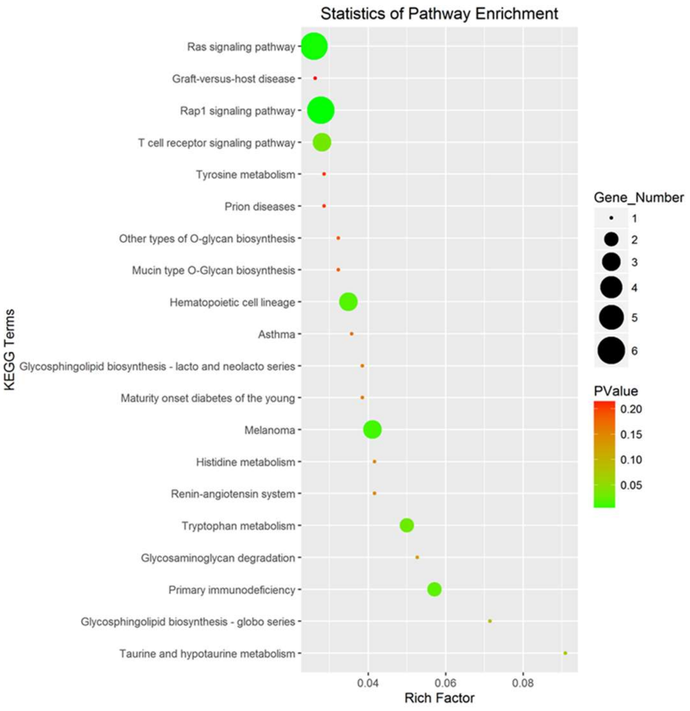

GSK

Fig. S6C

542

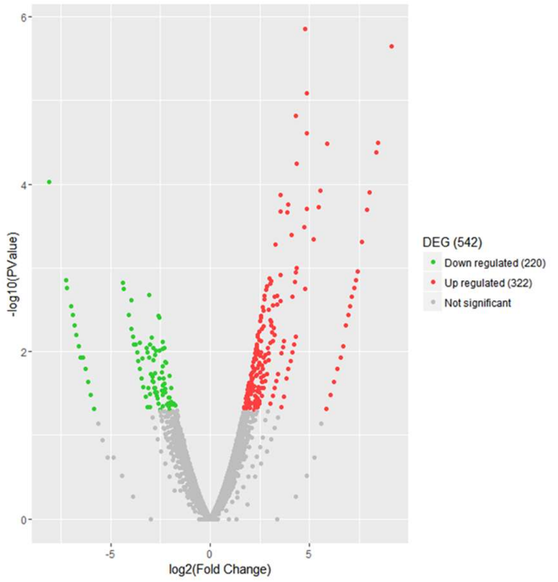

DEC

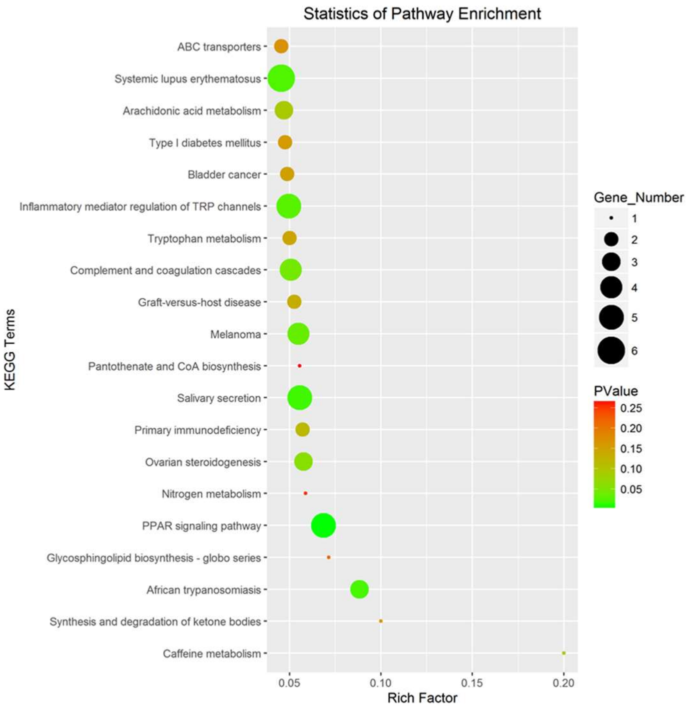

DEC

Fig. S6D

947

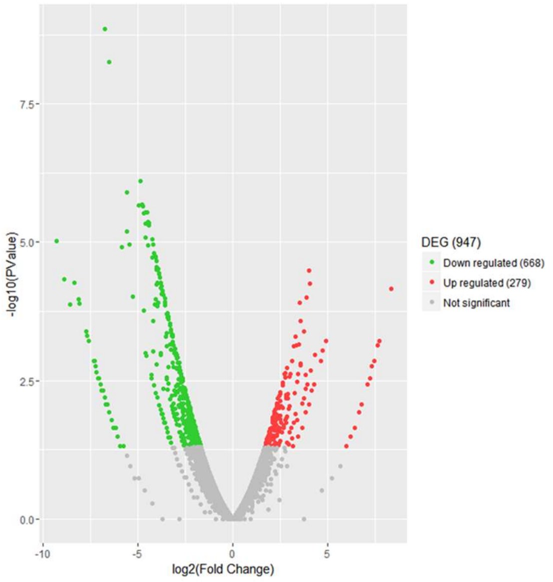

PAL

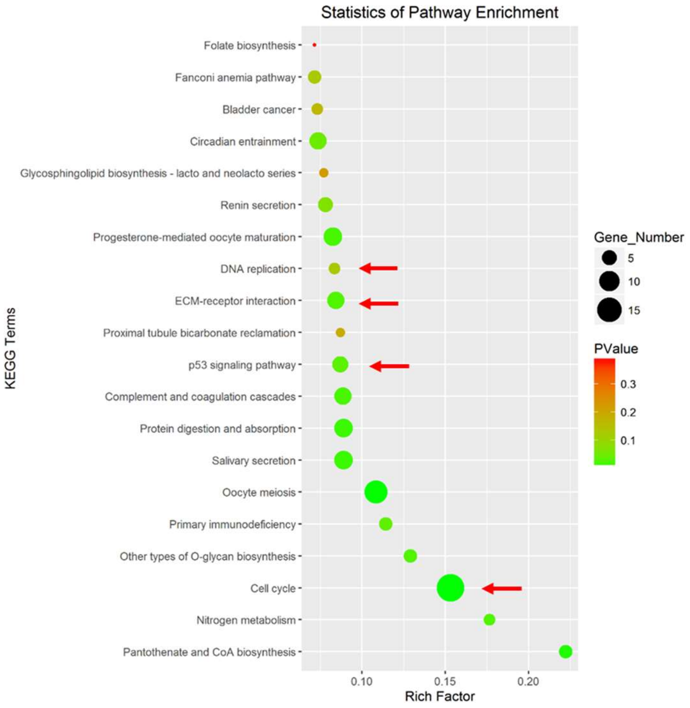

PAL

Fig. S6E

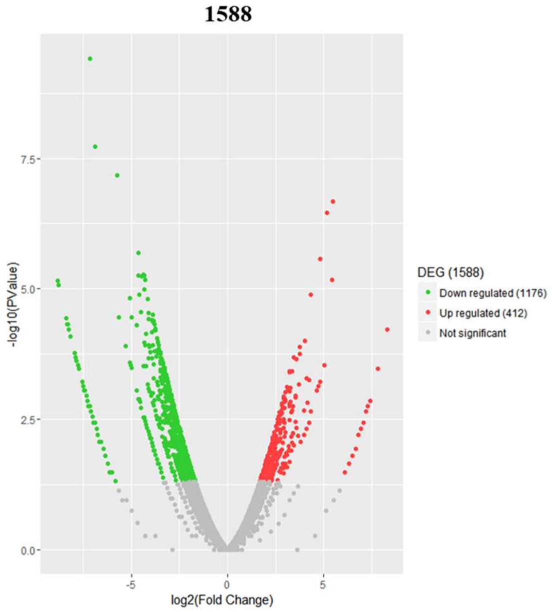

GEM + PAL

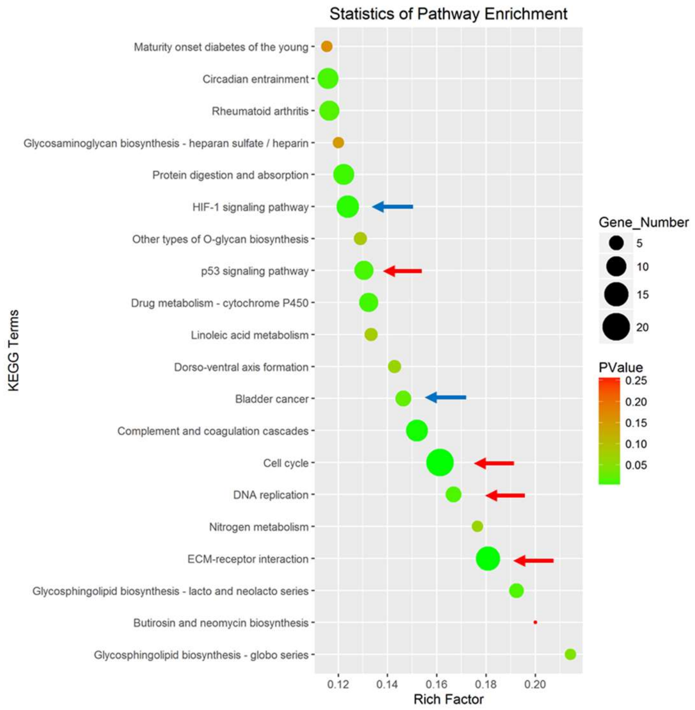

GEM + PAL

Fig. S6F.

KEGG cell cycle pathway

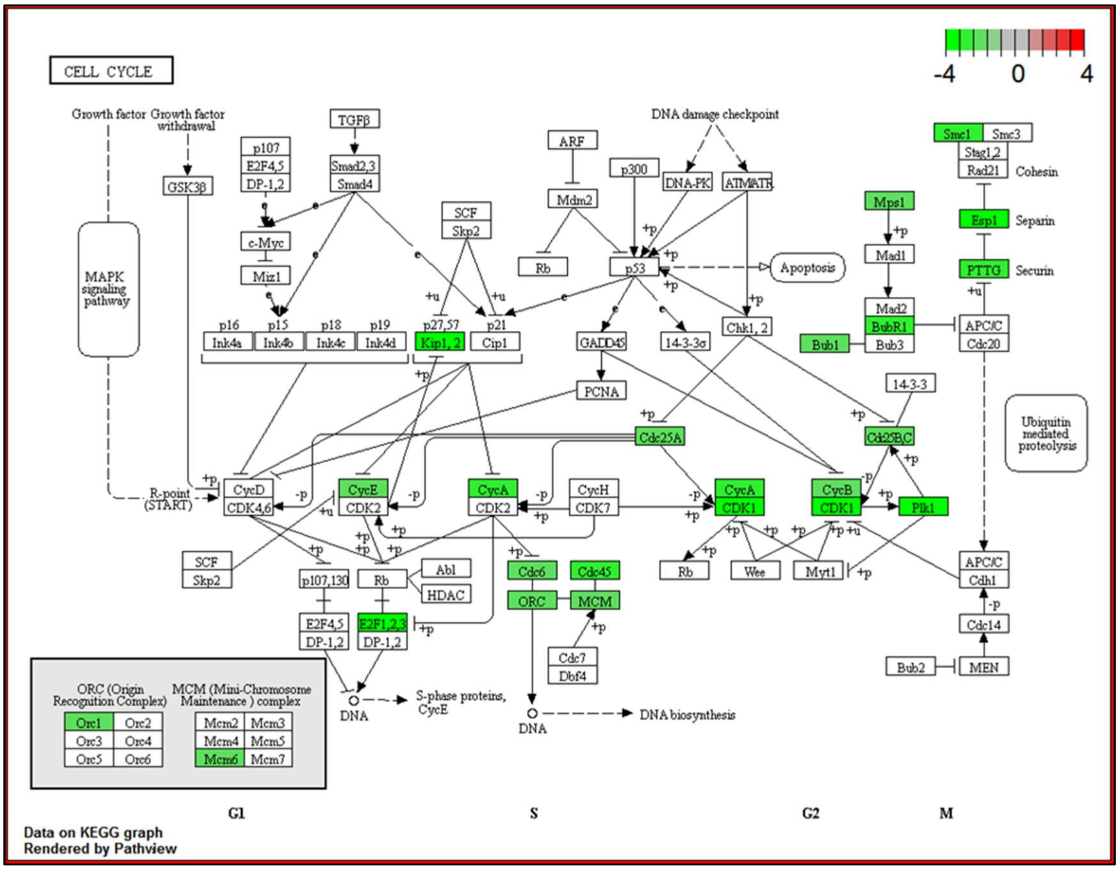

Fig. S6. Differentially expressed genes of PDX-Bone exposed to five different drugs.
