# Supplementary material for: Integrated genomic analyses in PDX model reveal a cyclin-dependent kinase inhibitor Palbociclib as a novel candidate drug for nasopharyngeal carcinoma
Source: J Exp Clin Cancer Res. 2018 Sep 20;37:233. doi: 10.1186/s13046-018-0873-5 (PMC6149192; doi:10.1186/s13046-018-0873-5)
Supplement: Supplementary file 12 — Table S5. Correlation between CNV detected by WES and Q-PCR. (PDF 298 kb) [file 13046_2018_873_MOESM12_ESM.pdf]

Table S5. Correlation between CNV detected by WES and Q-PCR

	WES/CNV				Q-PCR/CNV		
	CCND1	CDKN2A	RAD52		CCND1	CDKN2A	RAD52
PDX-ST	1.99	0.00	4.55		2.27	0.00	3.75
WBC-ST	1.97	2.06	2.11		1.94	2.12	2.15
PDX-LN	9.63	1.35	2.97		7.23	1.48	2.87
WBC-LN	2.04	2.03	1.99		2.07	2.73	2.40
PDX-LG	4.82	0.00	3.08		3.13	0.00	2.57
WBC-LG	2.02	1.88	2.04		1.53	1.99	2.21
PDX-LV	2.25	2.36	3.05		2.37	1.60	2.59
WBC-LV	1.97	2.03	1.86		1.44	1.78	1.68
PDX-B	6.00	0.00	2.00		3.13	0.00	1.22
WBC-B	2.00	2.00	2.00		1.55	1.71	1.77
				CORREL (r)	0.95	0.93	0.89
