# Supplementary material for: Integrated genomic analyses in PDX model reveal a cyclin-dependent kinase inhibitor Palbociclib as a novel candidate drug for nasopharyngeal carcinoma
Source: J Exp Clin Cancer Res. 2018 Sep 20;37:233. doi: 10.1186/s13046-018-0873-5 (PMC6149192; doi:10.1186/s13046-018-0873-5)
Supplement: Supplementary file 14 — Table S6. CNV of CCND1, p16 and RAD52 in the 11 NPC patients’ plasma with (A) high and (B) low EBV copy number. (PDF 783 kb) [file 13046_2018_873_MOESM14_ESM.pdf]

Table S6A. CNV of CCND1, p16 and RAD52 in the 11 NPC patients' plasma with high EBV copy no. (>5000/ml)

NPC	Plasma	EBV (copy no./ml)	log EBV (High)	CCND1	CDKN2A	RAD52	Ratio CCND1/CDKN2A
1	P7086	21,764	4.34	40.4	2.46	1.48	16.42
	P7333	477,336	5.68	10.28	0.89	1.87	11.55
2	p4736	7,112	3.85	14.1	2.77	2.43	5.09
	p4837	131,652	5.12	35.46	1.89	2.08	18.76
3	p4782	6,208	3.79	5.01	2.75	1.81	1.82
	p4831	36,140	4.56	35.06	5.35	2.31	6.55
4	p5246	6,180	3.79	6.37	2.34	1.85	2.72
	p5359	138,748	5.14	12.25	1.04	1.66	11.78
5	p4922	9,960	4	36.95	1.91	2.6	19.35
	p5034	19,508	4.29	18.14	1.34	1.45	13.54
6	p5681	7,072	3.85	13.11	1.99	2.74	6.59
	p5772	79,508	4.9	17.22	2.58	1.88	6.67
7	p5326	30,132	4.48	4.83	2.55	2.74	1.89
	p5733	300,128	5.48	105.31	1.65	1.94	63.82
8	p6560	5,068	3.7	8.84	2.11	3.57	4.19
	p7314	96,324	4.98	24.51	0.93	0.7	26.35
9	p6678	12,656	4.1	3.07	2.35	1.44	1.31
	p7096	479,596	5.68	13.88	0.68	2.35	20.41
10	p4366	10,688	4.03	6.21	1.41	2.78	4.40
	p4892	229,800	5.36	23.87	0.82	2.91	29.11
11	p4434	43,108	4.63	35.89	1.4	2.64	25.64
	p5233	1,458,344	6.16	19.93	0.93	3.86	21.43
			CORREL (r)	0.325	-0.488	0.056	0.576

Table S6B. CNV of CCND1, p16 and RAD52 in the 24 NPC patients' plasma with low EBV copy no. (<5000/ml)

Plasma	EBV (copy no./ml)	log EBV (Low)	CCND1	CDKN2A	RAD52	Ratio CCND1/CDKN2A
p5914	-	0	1.63	0.49	2.32	3.33
p6640	1308	3.12	1.48	2.27	2.25	0.65
p7917	584	2.77	5.08	1.54	3.41	3.30
p3435	27	1.43	1.8	2.08	1.81	0.87
p3490	107	2.03	1.84	1.56	2.21	1.18
p3619	613	2.79	4.62	2.18	4.43	2.12
p3715	141	2.15	2.5	1.97	2	1.27
p3686	-	0	1.75	2.32	1.87	0.75
p6391	304	2.48	1.73	1.15	1.65	1.50
p3787	175	2.24	1.77	1.5	2.28	1.18
p3962	16	1.2	2.41	1.3	1.9	1.85
p4012	2024	3.31	2.93	1.77	2.48	1.66
p4594	2904	3.46	2.71	1.45	1.6	1.87
p4315	193	2.29	2.05	1.87	1.72	1.10
p4608	-	0	1.82	1.71	1.91	1.06
p4780	48	1.68	0.47	2.1	2.12	0.22
p4826	1	0	1.9	2.33	2.04	0.82
p4317	78	1.89	3.56	1.96	1.78	1.82
p4550	-	0	1.81	1.99	1.99	0.91
p4924	312	2.49	1.59	1.61	2.03	0.99
p5249	-	0	1.72	1.58	1.95	1.09
p5646	104	2.02	1.99	1.83	2.14	1.09
p6780	-	0	1.74	1.25	2.22	1.39
p4974	68	1.83	1.68	1.44	1.64	1.17
		CORREL (r)	0.396	0.082	0.250	0.145
