# Supplementary material for: Integrated genomic analyses in PDX model reveal a cyclin-dependent kinase inhibitor Palbociclib as a novel candidate drug for nasopharyngeal carcinoma
Source: J Exp Clin Cancer Res. 2018 Sep 20;37:233. doi: 10.1186/s13046-018-0873-5 (PMC6149192; doi:10.1186/s13046-018-0873-5)
Supplement: Supplementary file 15 — Table S7. Immnunohistochemical (IHC) staining of cyclin D1 in 139 NPC tissues from year 2002 to 2016. (PDF 415 kb) [file 13046_2018_873_MOESM15_ESM.pdf]

Table S7. Immnunohistochemical (IHC) staining of cyclin D1 in 139 NPC tissues from year 2002 to 2016. Primary site: 33 samples; distant metastasis: 91 samples; and local regional recurrence: 15 samples.

	CCND1	Primary site (n=33)	Distant Metastasis (n=91)				Local regional recurrence (n=15)
			Lung	Bone	Liver	LN[1]+ soft tissue	T/N[2]
Total	n=139	33	38	13	27	13	15
Negative	(-) n=9	4	2	1	0	1	1
Positive	n=130	29/33 (87.9%)	36/38 (94.7%)	12/13 (92.3%)	27/27 (100%)	12/13 (92.3%)	14/15 (93.3%)
	+ n=14	1 (3.4%)	4 (11.1%)	4 (33.3%)	4 (14.8%)	0 (0%)	1 (7.1%)
	++ n=68	13 (44.8%)	20 (55.6%)	4 (33.3%)	15 (55.6%)	7 (58.3%)	9 (64.3%)
	+++ n=48	15 (51.7%)	12 (33.3%)	4 (33.3%)	8 (29.6%)	5 (41.7%)	4 (28.6%)

1. IHC staining: Negative: grade≦1 and cell population <5%; Positive: grade>1 and cell population≧5%.
2. LN[1]: lymph node; T/N[2]: primary site or local regional lymph node recurrence
