# Supplementary material for: Integrated genomic analyses in PDX model reveal a cyclin-dependent kinase inhibitor Palbociclib as a novel candidate drug for nasopharyngeal carcinoma
Source: J Exp Clin Cancer Res. 2018 Sep 20;37:233. doi: 10.1186/s13046-018-0873-5 (PMC6149192; doi:10.1186/s13046-018-0873-5)
Supplement: Supplementary file 16 — Table S8. Clinical characteristics of metastatic NPC patients with FFPE tissue cyclin D1 immnunohistochemical staining (2002–2016). (PDF 364 kb) [file 13046_2018_873_MOESM16_ESM.pdf]

Table S8. Clinical characteristics of metastatic NPC patients with FFPE tissue cyclin D1 immnunohistochemical staining (2002-2016)

	Total number (%)	CCND1 density, number (%)			P-value
		1 (N=16)	2 (N=46)	3 (N=29)	
Sex					
Male	80 (87.9)	16 (100)	39 (84.8)	25 (86.2)	0.259
Female	11 (12.1)	0 (0)	7 (15.2)	4 (13.8)	
Age					
Mean	51.1	50.8	52.8	48.8	0.343
Median	51.0	50	52.5	48.0	
Pathology					
undifferentiated	81 (89.0)	15 (93.8)	42 (91.3)	24 (82.8)	0.412
non-keratinising	10 (11.0)	1 (6.2)	4 (8.7)	5 (17.2)	
Metastatic site					
1	63 (69.2)	12 (75.0)	34 (73.9)	17 (58.6)	
2	23 (25.3)	3 (18.8)	11 (23.9)	9 (31.0)	0.542
3	3 (3.3)	1 (6.3)	0 (0)	2 (6.9)	
4	2 (2.2)	0 (0)	1 (2.2)	1 (3.4)	
Solitary					
1	35 (38.5)	8 (50.0)	20 (43.5)	7 (24.1)	0.091
≥ 2	56 (61.5)	8 (40.0)	26 (56.5)	22 (75.9)	
Chemotherapy					
¥ G+P	43 (47.3)	9 (56.3)	24 (52.2)	10 (34.5)	0.239
# P/U/L+(B/M)	48 (52.7)	7 (43.8)	22 (47.8)	19 (65.5)	
**Overall survival**					
Month (median)	24.0	52.0	26.0	15.0	**< 0.001**
Month (95% CI)	19.9 – 28.1	8.6 – 95.4	21.7 – 30.3	7.5 – 22.5	
**EBV*** (copies/ml) mean	23737	3221 (N=13)	10301 (N=43)	57515 (N=25)	**0.046**

¥ G+P: gemcitabine+cisplatin

P/U/L+(B/M): cisplatin+UFT+calcium folinate + (bleomycin or mitomycin-c)

* Among 91 metastatic NPC patients only 81 had plasma EBV DNA (copies/ml) data (patient's blood was collected after receiving metastatic site tissue proof).
