# Supplementary material for: Integrated genomic analyses in PDX model reveal a cyclin-dependent kinase inhibitor Palbociclib as a novel candidate drug for nasopharyngeal carcinoma
Source: J Exp Clin Cancer Res. 2018 Sep 20;37:233. doi: 10.1186/s13046-018-0873-5 (PMC6149192; doi:10.1186/s13046-018-0873-5)
Supplement: Supplementary file 17 — Table S9. PDX tumor growth during drug treatment (A) PDX-Bone tumor volume (mm3) and tumor weight (g), and (B) PDX-Bone mice body weight (g) in drug screening; (C) PDX-LN tumor volume (mm3) and tumor weight (g), and (D) PDX-LN mice body weight (g) in drug screening. (PDF 241 kb) [file 13046_2018_873_MOESM17_ESM.pdf]

Table S9. (A) PDX-Bone tumor volume (mm3) and tumor weight (g) in drug screening

PDX-Bone (A) Tumor volume (mm^3) and tumor weight (g)

group	ear tag number	day 0	day 4	day 7	day 11	day 14	day 18	day 21	**Vol. Fold**	**Avg (Fold)**	**SD**	**T-test**	**Tumor (g)**	**Avg**	**SD**	**T-test**
DMSO	DMSO1	127.3	225.2	293.7	502.6	822.2	1055.4	1592	12.50589	**15.45**	3.774176		1.421	**1.15833**	0.2822	
	DMSO2	74.7	95.1	127.7	235.5	405.4	772.9	1056.3	14.14056				0.86			
	DMSO3	73.6	138.9	205.8	414.1	601.3	1075.7	1450.3	19.70516				1.194			
Gemcitabine	GEM1	51.7	74.4	91.3	98.3	108.6	128.7	155.5	3.007737	**3.96**	3.192214	0.01577	0.135	**0.214**	0.20901	0.00961
	GEM2	83.9	146.9	193	303.8	331.3	395.6	630.6	7.516091			*	0.451			**
	GEM3	56.2	57.7	60.9	64.5	57.5	63.8	75.7	1.346975				0.056			
GSK126	GSK1	76	118.6	125.1	178	273.6	364.9	383.7	5.048684	**7.19**	1.990564	0.02851	0.348	**0.45633**	0.13531	0.01776
	GSK2	56.3	78.9	118	176.8	244.6	381.4	505.7	8.982238			*	0.413			*
	GSK3	96	169.5	218.4	339.1	375.7	510.5	724.5	7.546875				0.608			
Decitabine	1-Dec	71.8	126.1	211.2	217.8	272.2	346.8	378.3	5.268802	**7.35**	2.394845	0.03491	0.286	**0.40167**	0.20818	0.02017
	2-Dec	80	136.5	187.2	316.7	470.4	646.4	797.5	9.96875			*	0.642			*
	3-Dec	52.1	61.3	92.4	121	152.4	227.4	355.3	6.819578				0.277			
Palbociclib	PAL1	98.2	143	172.2	200.2	224.1	215.9	196.5	2.001018	**1.60**	0.757198	0.00338	0.149	**0.12367**	0.04917	0.00333
	PAL2	71.6	107.3	100	99	85.1	49.5	52.1	0.727654			**	0.067			**
	PAL3	94.3	166.5	191	204.8	195	176.3	195.6	2.074231				0.155			
Gem.+Pal	GEM+PAL1	62.6	82.3	75.9	76.4	65.7	59.5	47.6	0.760383	**0.85**	0.35733	0.00262	0.025	**0.037**	0.01908	0.00236
	GEM+PAL2	51.7	55.2	71.1	62.1	42	35.3	27.9	0.539652			**	0.027			**
	GEM+PAL3	66.2	145.7	132.2	109.8	103.5	106.1	82	1.238671				0.059			

Table S9. (B) PDX-Bone mice body weight (g) in drug screening

PDX-Bone (B) Mice body weight (g)

group	ear tag number	day 0	day 4	day 7	day 11	day 14	day 18	day 21	**Avg (g)**	**SD**
DMSO	DMSO1	27.9	28.5	28.2	29.1	29.5	30.7	30.7	30.47	0.321455
	DMSO2	27.7	27.9	28.7	28.6	28.6	29.5	30.1		
	DMSO3	27.3	26.6	28.5	28.7	29.4	30.2	30.6		
Gemcitabine	GEM1	27.7	27.3	27.7	28	28.2	28	28.2	28.60	1.153256
	GEM2	28.5	28.3	28.7	28.5	29.3	30.4	29.9		
	GEM3	27	26.6	27	27.1	27.2	26.9	27.7		
GSK126	GSK1	25.1	25.5	25.4	25.6	24.8	25.8	24.2	24.90	0.7
	GSK2	25.7	25.8	25.5	25.5	25.8	25.7	24.9		
	GSK3	26.3	27	26.3	27	24.8	26.1	25.6		
Decitabine	1-Dec	26.9	26.9	26.5	27.4	27	27.7	24.4	26.67	2.354428
	2-Dec	27.2	28.3	27.6	28.3	28.2	30.3	29.1		
	3-Dec	26.4	26.7	26.4	27.1	25.8	27.7	26.5		
Palbociclib	PAL1	28.3	28.7	28.3	29.2	29.2	30.2	29.1	25.70	3.616628
	PAL2	25.1	25.5	25.3	26.6	26.1	21.7	21.9		
	PAL3	29	28.7	28.6	29.2	28.3	25.6	26.1		
Gem.+Pal.	GEM+PAL1	27.8	28.1	28.6	28.6	28.7	29.1	25.4	26.83	1.266228
	GEM+PAL2	28	28.1	26.9	26.6	26.8	28.6	27.8		
	GEM+PAL3	25.7	26.7	26.2	25.7	24.9	27.4	27.3		

Table S9. (C) PDX-LN tumor volume (mm^3) and tumor weight (g) in drug screening

PDX-LN (A) Tumor volume (mm^3) and tumor weight (g)																
Group	ear tag number	day 0	day7	day14	day21	day 28	day 35	Vol. Fold	Avg (Fold)	SD	T-test	Tumor (g)	Avg	SD	T-test
DMSO	DSMO1	451.4	632.8	876.3	1243.1	1401.9	1,999.00	4.428445	**3.53**	0.78313		1.258	**0.82533**	0.37567	
	DSMO2	348.9	455.9	598.5	752.6	1,010.60	1,094.80	3.137862				0.636			
	DSMO3	301.6	473.4	608.7	758	883.4	909.2	3.014589				0.582			
GSK126	GSK1	296.7	381.5	421.2	669.6	1,015.90	1,209.40	4.076171	**2.44**	1.48725	0.32581	0.707	**0.46867**	0.25196	0.24378
	GSK2	342.6	501.4	554.2	655.5	750.3	712	2.078225				0.494			
	GSK3	223.9	194.2	232.2	211.7	181.3	261.7	1.168825				0.205			
Gemcitabine	GEM1	490.7	620.1	145.7	99.3	64.8	49.8	0.101488	**0.09**	0.01922	0.00161	0.0258	**0.01687**	0.00786	0.02036
	GEM2	427.6	327.5	101.3	57.4	42.4	29.4	0.068756			**	0.011			*
	GEM3	493.3	359.4	114.7	64.7	55.5	50.6	0.102574				0.0138			
Palbociclib	PAL1	323.6	370.1	303	213.5	195.5	183.7	0.567676	**0.48**	0.07994	0.00257	0.135	**0.12933**	0.06768	0.03425
	PAL2	191.6	187.1	173.8	165.1	126.8	79.5	0.414927			**	0.059			*
	PAL3	536.2	507.8	497	383.4	316.8	241.5	0.450392				0.194			

Table S9. (D) PDX-LN mice body weight (g) in drug screening

PDX-LN (B) Mice body weight (g)									
Group	ear tag number	day 0	day7	day14	day21	day 28	day 35	Avg (g)	SD
DMSO	DSMO1	31.4	31	31.4	32.5	32.6	32	32.27	0.9291573
	DSMO2	30.4	30.2	30.9	31	30.9	31.5		
	DSMO3	32.8	33.7	33.6	33	33.8	33.3		
GSK126	GSK1	30.3	28.7	28	28.9	30.3	29.9	26.53	3.0369941
	GSK2	29.5	29.4	28.5	28.2	27.5	24		
	GSK3	31.9	31.3	28.7	27.6	27.4	25.7		
Gemcitabine	GEM1	31.2	31.4	30.7	31.2	29.9	28.6	29.90	1.2529964
	GEM2	30.8	31	31.9	30.6	31	31.1		
	GEM3	30.6	301	29.8	29.6	30	30		
Palbociclib	PAL1	29.5	25.7	26.4	27.6	28.5	28.7	27.03	1.942507
	PAL2	30.2	23.5	28.3	28.9	29.1	24.9		
	PAL3	29.6	25.9	27	23.8	25.5	27.5		
